# Ending the HIV Epidemic in Metropolitan Atlanta: a mixed‐methods study to support the local HIV/AIDS response

**DOI:** 10.1002/jia2.26322

**Published:** 2024-07-22

**Authors:** Micah Piske, Bohdan Nosyk, Justin C. Smith, Bianca Yeung, Benjamin Enns, Xiao Zang, Patrick S. Sullivan, Wendy S. Armstrong, Melanie A. Thompson, Gaea Daniel, Carlos del Rio

**Affiliations:** ^1^ Centre for Advancing Health Outcomes St. Paul's Hospital Vancouver British Columbia Canada; ^2^ Faculty of Health Sciences Simon Fraser University Burnaby British Columbia Canada; ^3^ Positive Impact Health Centers Atlanta Georgia USA; ^4^ Harvard T.H. Chan School of Public Health Boston Massachusetts USA; ^5^ Division of Health Policy and Management School of Public Health University of Minnesota Minneapolis Minnesota USA; ^6^ Department of Epidemiology Emory University Rollins School of Public Health Atlanta Georgia USA; ^7^ Division of Infectious Diseases Department of Medicine Emory University School of Medicine Atlanta Georgia USA; ^8^ Grady Health System Atlanta Georgia USA; ^9^ Thacker & Thompson Atlanta Georgia USA; ^10^ Nell Hodgson Woodruff School of Nursing Emory University Atlanta Georgia USA

**Keywords:** HIV epidemiology, HIV prevention, testing, PrEP, structural drivers, public health

## Abstract

**Introduction:**

Four counties within the Atlanta, Georgia 20‐county eligible metropolitan area (EMA) are currently prioritized by the US “Ending the HIV Epidemic” (EHE) initiative which aims for a 90% reduction in HIV incidence by 2030. Disparities driving Atlanta's HIV epidemic warrant an examination of local service availability, unmet needs and organizational capacity to reach EHE targets. We conducted a mixed‐methods evaluation of the Atlanta EMA to examine geographic HIV epidemiology and distribution of services, service needs and organization infrastructure for each pillar of the EHE initiative.

**Methods:**

We collected 2021 county‐level data (during June 2022), from multiple sources including: AIDSVu (HIV prevalence and new diagnoses), the Centers for Disease Control and Prevention web‐based tools (HIV testing and pre‐exposure prophylaxis [PrEP] locations) and the Georgia Department of Public Health (HIV testing, PrEP screenings, viral suppression and partner service interviews). We additionally distributed an online survey to key local stakeholders working at major HIV care agencies across the EMA to assess the availability of services, unmet needs and organization infrastructure (June−December 2022). The Organizational Readiness for Implementing Change questionnaire assessed the organization climate for services in need of scale‐up or implementation.

**Results:**

We found racial/ethnic and geographic disparities in HIV disease burden and service availability across the EMA—particularly for HIV testing and PrEP in the EMA's southern counties. Five counties not currently prioritized by EHE (Clayton, Douglas, Henry, Newton and Rockdale) accounted for 16% of the EMA's new diagnoses, but <9% of its 177 testing sites and <7% of its 130 PrEP sites. Survey respondents (*N* = 48; 42% health agency managers/directors) reported high unmet need for HIV self‐testing kits, mobile clinic testing, HIV case management, peer outreach and navigation, integrated care, housing support and transportation services. Respondents highlighted insufficient existing staffing and infrastructure to facilitate the necessary expansion of services, and the need to reduce inequities and address intersectional stigma.

**Conclusions:**

Service delivery across all EHE pillars must substantially expand to reach national goals and address HIV disparities in metro Atlanta. High‐resolution geographic data on HIV epidemiology and service delivery with community input can provide targeted guidance to support local EHE efforts.

## 1 INTRODUCTION

The Atlanta, Georgia, Metropolitan Statistical Area (MSA) is the eighth largest in the United States and one of the country's fastest‐growing metropolitan areas [1, 2]. The Ryan White HIV/AIDS Program (RWHAP) eligible metropolitan area (EMA) of Atlanta (a jurisdiction designated for federal HIV funding allocation) is comprised of 20 counties, four of which are among the 48 counties currently prioritized by the US Ending the HIV Epidemic (EHE) initiative to reduce HIV infections at least 90% by 2030 through four pillars: HIV diagnosis, prevention, treatment and response [3, 4]. In 2021, metropolitan Atlanta recorded the third highest HIV diagnoses rate among US MSAs (25.4 diagnoses per 100,000 people) [5]. Racial, ethnic, geographic, sexual and gender minority disparities in the impact of HIV continue to widen nationally and illustrate an epidemic perpetuated by long‐standing social injustices and structural racism—particularly in the South where Black people account for more of the overall population than other regions of the country (e.g. 31% in Georgia vs. 13% nationally) and for six of every 10 new HIV diagnoses [6, 7]. HIV prevalence concentrated within Atlanta's downtown core is estimated at 1.34%, meeting the UNAIDS definition of a “generalized epidemic” [8].

Georgia remains one of 10 states yet to fully expand Medicaid coverage under the Affordable Care Act despite that Medicaid expansions are linked to reductions in mortality [9, 10, 11] and net savings on state budgets [12, 13]. Furthermore, Congressional EHE appropriations fell below original budget requests in 2021 ($404M funded, $716M requested) and 2022 ($473M funded, $670M requested) [14, 15]. Although cities and states do not provide most of the funding for HIV care and prevention, local public health departments and hospitals are critical decision‐makers in securing funding and allocating resources. This disconnect between funding and decision‐making can create challenges when the costs and benefits of improved HIV care are unevenly distributed across payors, and when funding is reliant on discretionary appropriations that can be diverted to other priorities [16].

Many of the needs and proposed activities noted in Georgia's 2021−2025 EHE plan were highlighted in a series of prior reports released by the Fulton County Task Force on HIV/AIDS, listing a range of actionable recommendations across sectors to support HIV/AIDS control in Atlanta [17]. Although many of these efforts have stalled as a result of the COVID‐19 response [18, 19], Georgia has repealed punitive HIV‐specific provisions of the criminal code [20] and legalized syringe service programmes (SSPs) [21]. Despite these advances, in 2021, 2371 people in Georgia were diagnosed with HIV, a decrease of <3% from 2439 persons diagnosed in 2019 [22, 23].

A series of prior modelling studies demonstrated increasing the scale of 10 evidence‐based interventions for HIV testing (screening reminders and rapid testing), prevention (pre‐exposure prophylaxis [PrEP], syringe services, medications for opioid use disorder) and treatment (rapid initiation, case management and care coordination) in Atlanta would reduce HIV incidence 32% by 2030 while providing long‐term cost savings [24]. Furthermore, equitable delivery of these interventions reducing racial/ethnic disparities in care access could prompt a 69% incidence reduction, with long‐term savings of $579.8M over 20 years [25]. Given the analysis was executed at the EMA level, the means of how and where to direct these resources requires data on geographic HIV epidemiology, service availability and supporting healthcare infrastructure. These recommendations must be further reconciled with community needs and operationalized with feasible implementation and financing strategies.

The Association of State and Territorial Health Officials profile survey represents the only comprehensive national data source regarding state public health agency activities, workforce capacity and composition [26]; however, the scope of clinical services assessed cover a range of disease areas without specific interventions assessed for HIV. While successful intervention implementation requires readiness for care agency staff to adopt these changes [27], there is a lack of supporting data in these areas to inform EHE implementation at the local level.

The COVID‐19 pandemic fundamentally altered the public health landscape, with changes in funding, service delivery and human resources impacting HIV/AIDS care and outcomes [28, 29]. As the health system continues its slow recovery, careful consideration of metropolitan Atlanta's current capacity and service availability across each of the pillars of the EHE initiative is needed to galvanize efforts to reach EHE targets. Using the Atlanta RWHAP EMA as a focal region, we executed a mixed‐methods evaluation of the breadth, capacity and geographic distribution of metropolitan Atlanta's HIV services as well as organizational readiness for service expansion according to local stakeholders.

## 2 METHODS

### 2.1 HIV epidemiology and health services in the Atlanta EMA

We conducted a comprehensive examination of geographic HIV epidemiology and services offered in the RWHAP Part A EMA of Atlanta (Supplementary Appendix A1) during June 2022 for each pillar of the EHE strategy. We extracted data from multiple sources: county‐level 2021 AIDSVu data on HIV prevalence, new diagnoses, social determinants of health and PrEP‐to‐Need Ratio (i.e. PrEP users in 2021 to new HIV diagnoses in 2020) [30]; the US Centers for Disease Control and Prevention (CDC) National Prevention Information Network (health agencies and service fee information) [31]; CDC Get Tested website (HIV testing locations and costs) [32]; CDC Care and Prevention in the United States (CAPUS) tool (PrEP locations) [33]; CDC‐funded HIV testing reports (partner services interviews) [34]; and the North American Syringe Exchange Network website (SSP locations) [35]. We additionally obtained 2021 county‐level data from the Georgia Department of Public Health (GDPH) on GDPH‐funded testing events, PrEP eligibility screenings, people living with HIV (PLHIV) virally suppressed and persons newly diagnosed interviewed for partner services [36]. We determined the number and location of sites providing HIV testing, PrEP and SSPs (as of July 2022), and summarized by county: HIV testing sites per 100,000 population; availability of rapid HIV tests (results within 30 minutes onsite) and self‐testing kits; facility type; and availability of free HIV tests (no out‐of‐pocket cost regardless of insurance coverage).

### 2.2 Community stakeholder survey

In parallel (during June−December 2022), we distributed an online survey to key local stakeholders including physicians (infectious disease and HIV specialists, family and internal medicine), nurse practitioners, physician assistants, other health agency staff (including managers and administrators), as well as community advocates and PLHIV working within major HIV care agencies and community‐based organizations within the EMA. HIV care agencies were identified from a list of major HIV care providers in 2021, obtained from the Georgia Medical Monitoring Project [37] and through consultation and referrals from local stakeholders in the EHE planning network. We recruited stakeholders via snowball sampling, beginning with referrals from survey respondents.

We adapted several standardized assessments including the Association of State and Territorial Health Officials (ASTHO) Profile Survey [38] and the Organizational Readiness for Implementing Change (ORIC) survey [39] to assess health agency activities and unmet needs within the community served (HIV treatment, prevention, diagnosis, partner services, integrated care and services addressing social determinants of health); PrEP prescribing experience; perceived organization implementation climate for scaling‐up or implementing priority interventions; and organizational infrastructure. The 68‐item questionnaire (average 15 minutes completion time) assessed respondent demographics; client demographics (if applicable); service availability, perceived unmet need by service (5‐point Likert scale from lowest to highest unmet need), as well as specific services and/or resources needed to address unmet needs in the community (open‐ended free text). For prescribers, six items measured comfort and experience prescribing PrEP [40] (PrEP familiarity, discussing sexual health, determining indication, comfort prescribing, experience) using a 5‐point Likert scale measuring agreement. Organization implementation climate for service expansion or implementation was assessed by the 12‐item ORIC tool with 5‐point Likert scales for agreement relating to perceived organizational commitment, determination, motivation and sustainment for a selected priority service. Respondents were additionally asked to describe potential implementation barriers (open‐ended free text). Finally, an optional supplementary 27‐item questionnaire assessed organization infrastructure (HIV service staff including peer outreach workers, vacancies and salary ranges). A detailed description of survey components, measures and respondents is provided in Supplementary Table A1.

### 2.3 Data analysis

#### 2.3.1 Geographic distribution of HIV care agencies

We plotted HIV testing (and sites with free tests), PrEP and SSP locations throughout the EMA on a choropleth map illustrating rates of PLHIV per 100,000 population, EHE jurisdiction boundaries and rates of testing sites by county. Maps were created in ArcGIS (Esri, Redlands, CA) with shapefiles from the Atlanta Regional Commission and Georgia Department of Transportation [41, 42]. Geographic data informed the development of survey questions (e.g. scope and service types) and interpretation of responses.

#### 2.3.2 Quantitative survey responses

First, we presented characteristics of survey respondents and their clients/patients (if applicable). Second, we presented responses on the availability of services within agencies, perceived unmet need rating within the community and percentage of responses indicating high/highest unmet need (Likert scale responses ≥4). Third, we presented ratings on PrEP prescribing experience and the percentage of responses indicating agreement (Likert scale responses ≥4). We used Mann−Whitney U tests to assess the difference in ratings between HIV specialists a other prescribers. Fourth, we presented summarized and total ORIC ratings by selected priority service and percentage of respondents indicating agreement by statement. Finally, we presented the reported number of employees, full‐time equivalents (FTEs), vacancies and salary ranges by position.

#### 2.3.3 Qualitative survey responses

All participants provided at least one free‐text response ranging in length from 2 to 3 sentences (on average) up to several paragraphs. After a careful review of these responses, we conducted a thematic content analysis to comprehensively examine, capture and summarize the voices of participants relating to unique resource needs and implementation barriers not otherwise captured in the questionnaire. Optional discussions (*N* = 13) were held over Zoom with representatives to introduce the study, answer questions, confirm suitability and allow space for participants to provide relevant contextual information. Where contextual/service delivery information was provided, two research team members facilitating the discussions took notes and reviewed them for comprehensiveness and agreement.

Each free‐text response was coded manually through inductively identifying unique codes within the data to highlight key concepts and deductively based on discussions held with respondents (independently by two research team members). The iterative approach was defined by grouping comments by topic and analysing each response until thematic saturation was reached. Code definitions and examples were discussed at research team meetings for consensus. A total of 32 unique codes were identified and analysed for consistency and agreement (Supplementary Appendix B). Key themes were presented according to activity by EHE pillar with representative quotations. These responses additionally informed the interpretation of the quantitative responses and the geographic data on service locations. We, therefore, conducted a mixed‐methods (parallel) analysis examining both quantitative and qualitative data, drawing inferences from their integration to answer the research questions and inform recommendations [43].

### 2.4 Ethical considerations

The study was approved by the Simon Fraser University and Providence Health Care research ethics boards (H16‐00652) and all participants provided informed consent.

## 3 RESULTS

### 3.1 HIV epidemiology in the Atlanta EMA

The Atlanta EMA ranged in population from 34,524 (Pickens) to 1,081,158 (Fulton) in 2022 (Table 1). The four EHE counties accounted for 77% of new HIV diagnoses, and 80% of PLHIV in the EMA. Despite accounting for only 32% of Georgia's population, Black/African Americans comprised over 68% of Georgia's PLHIV and 71% of new HIV diagnoses in 2021. These disparities were consistent across the EMA—Black/African Americans accounted for 36% of the population yet 69% of the EMA's PLHIV and 72% of new HIV diagnoses. Counties with the highest income inequality, and with greater proportions of people living with severe housing cost burdens recorded higher rates of new diagnoses and HIV prevalence and the lowest rates of viral suppression. Rates of new diagnoses in EHE counties ranged from 21 (Gwinnett) to 58 (Fulton) individuals per 100,000. Several EMA counties outside of the EHE jurisdictions recorded new diagnosis rates greater than in EHE counties, including Clayton (48 per 100,000), Douglas (30 per 100,000), Newton (24 per 100,000) and Rockdale (46 per 100,000).

**Table 1:** Demographic population characteristics in Georgia, US, by county in 2021/22

		Ending the HIV Epidemic (EHE) counties				
	State of Georgia	Cobb	DeKalb	Fulton	Gwinnett	Other non‐EHE counties in the Atlanta EMA
*Total population, 2022* ^a^	10,891,679	768,757	762,728	1,081,158	975,029	2,429,219
Non‐Hispanic White or other	6,272,520 (58%)	4,544,626 (58%)	288,419 (38%)	528,476(49%)	473,394 (49%)	1,436,599 (59%)
Black/African American	3,492,925 (32%)	217,390 (28%)	408,421(53%)	473,839(44%)	284,020 (29%)	760,054 (31%)
Hispanic/Latinx	1126,234 (10%)	106,741 (14%)	65,888 (9%)	78,843 (7%)	217,615 (22%)	232,566 (10%)
*Projected population growth by 2030*						
(% Growth from 2022)^a^	8%	8%	4%	10%	9%	13%
% Non‐Hispanic White or other	5%	3%	2%	7%	3%	9%
% Black/African American	10%	13%	4%	12%	16%	19%
% Hispanic/Latinx	17%	18%	16%	13%	15%	19%
*Other socio ‐ demographic metrics 2021* ^b^, ^c^						
Drug overdose deaths per 100,000	22.1	21.8	21.4	23.9	17.1	13.5−43.3
Income inequality (Gini coefficient)	0.48	0.45	0.49	0.53	0.44	0.36−0.55
% Living in poverty	13.9	8.6	13.5	12.9	10..5	5.4−17.3
% Uninsured	15.1	14.3	15.1	11.2	17.5	9.3−20
% Living with severe housing cost burden^d^	13.7	12	15.9	16.3	15.2	9.3−17.8
*New HIV d iagnoses 2021* ^c^	***2371***	152	342	525	165	344
Rate of new diagnoses per 100,000	26	24	54	58	21	5−48
% Non‐Hispanic White	14.6	15.1	7.3	10.7	12.7	16.36
% Black/African American	71.3	62.5	78.9	78.3	52.1	70.9
% Hispanic/Latinx	10.3	18.4	9.9	7.6	27.9	−^e^
*People living with HIV 2021* ^c^	59,422	3653	9140	16,384	3347	8149
Rate of PLHIV per 100,000	657	565	1443	1802	423	121−1049
% Non‐Hispanic White	24.5	32.9	16.9	21.4	26.5	16.3
% Black/African American	68.5	60.6	73.1	71.8	57.4	66.3
% Hispanic/Latinx	8.4	12.6	8.2	6.7	19.9	7.4
% PLHIV virally suppressed	62.1	66.6	64	62.4	67.1	61.4−73.8
*PrEP‐to‐Need Ratio 2021* ^†^, ^c^	5.01	5.23	6.07	7.69	5.8	2.53−20.5

Abbreviations: EMA, eligible metropolitan area (see Supplementary Section A1 for detail); PLHIV, people living with HIV; PrEP, pre‐exposure prophylaxis.

^†^ Defined as the ratio of the number of PrEP users in 2021 to the number of people newly diagnosed with HIV in 2020, measuring whether PrEP use reflects the need for HIV prevention (a lower PrEP‐to‐Need‐Ratio indicates more unmet need).

^a^ Data source: State of Georgia Governor's Office of Planning and Budget, State and County Projections by race, 2022−2060.

^b^ Drug overdose deaths data source: Georgia Department of Public Health 2003−2022 Online Analytical Statistical Information System (OASIS) Drug Overdoses Mortality Web Query Tool.

^c^ Data source: Sullivan PS, Woodyatt C, Koski C, Pembleton E, McGuinness P, Taussig J, Ricca A, Luisi N, Mokotoff E, Benbow N, Castel AD. A data visualization and dissemination resource to support HIV prevention and care at the local level: analysis and uses of the AIDSVu Public Data Resource. Journal of Medical Internet Research. 2020;22(10):e23173.

^d^ Defined as percent of people who spend over 50% of their income on housing.

^e^ Data supressed due to small cell sizes.

### 3.2 Survey respondents and client demographics

From a total of 148 invited participants, we collected 48 (32%) completed surveys representing 25 organizations across the EMA, including 26 participants (54%) from clinical settings, 17 (35%) from community‐based organizations and 5 (10%) representing public health departments or other organizations (Table 2). Thirty‐nine (81%) respondents were physicians and/or in other health agency roles and five self‐reported as a community advocate and/or person living with HIV. Over half (55%) identified as female, 35% were Black/African American, 30% White, 15% Asian, <10% Hispanic/Latinx and <10% multiracial or other. Care providers reported a median HIV caseload make‐up of 73% Black/African American, 15% White and 7% Hispanic/Latinx clients; 70% men who have sex with men, 20% heterosexuals and 5% people who inject drugs.

**Table 2:** Survey respondent characteristics and care provider/health agency staff‐reported client characteristics

Respondent characteristics (*N* = 48)	*N* (%)
Organization/facility type:	
Primary care clinic or hospital	26 (54)
Community‐based organization	17 (35)
Public health department or other organization	5 (10)
Organization (primary) location	
EHE jurisdiction (Cobb, DeKalb, Fulton or Gwinnett County)	39 (81)
Other jurisdiction in the metropolitan area	9 (19)
Role/position ^a^ :	
Physician	19 (40)
Infectious disease board‐certified and/or HIVMA/AAHIV‐S	13
Family/internal medicine board‐certified or other	8
Nurse, nurse practitioner, physician assistant or other care provider role	6 (13)
Health agency employee (Manager, Administrator or other role)^b^	20 (42)
Community advocate and/or person living with HIV/AIDS	5 (10)
Gender:	
Female	(55)
Male	(40)
Non‐conforming/non‐binary or other	(≤ 5)
Race/Ethnicity:	
Black/African American/Afro‐Caribbean	16 (35)
White	15 (30)
Asian	8 (15)
Hispanic/Latinx	(<10)
Two or more races or other	(<10)
**Care provider or agency staff‐reported client characteristics (*N* = 45)**	**No**.
No. clients/patients with HIV in the past 12 months (*N* [%]):	
None	11 (24)
1−20	11 (24)
21−50	2 (4)
51−199	11 (24)
≥ 200	10 (22)
No. hours devoted to direct patient care and client support per week (*N* [%]):	
None	17 (38)
1−20	6 (13)
21−30	7 (16)
31−40	4 (9)
≥ 40	11 (24)
Racial/ethnic makeup of clients with HIV in the past 12 months (Median % [Q1, Q3]):	
Black/African American	73 (54, 90)
Hispanic/Latinx	7 (3, 10)
White	15 (10, 30)
Other	0 (0, 2)
Characteristics of clients with HIV in the past 12 months (Median % [Q1, Q3]):	
Men who have sex with men	70 (53, 88)
Heterosexual	20 (10, 39)
Injection drug user	5 (2, 10)
Other	0 (0, 0.5)
Insurance status of clients with HIV in the past 12 months (Median % [Q1, Q3]):	
Private—(through employer)	30 (10, 60)
Private—other	10 (5, 19)
Public—Medicaid	20 (10, 31)
Public—Medicare	20 (10, 20)
Other coverage	5 (0, 25)
Uninsured	40 (5, 70)

Abbreviation: EHE, Ending the HIV Epidemic initiative.

^a^ More than one role could apply for respondents.

^b^ Includes public health department and community‐based organization staff. A total of 25 distinct organizations in the Atlanta metropolitan area were represented in these responses.

For staff‐reported client characteristics, respondents were asked the following questions in order if applicable to them: 1. How many patients or clients with HIV have you provided continuous and direct care or support for in the past 12 months?; 2. How many hours do you devote to patient care or client support per week; 3. Please estimate the racial/ethnic makeup; proportion of the following risk groups; and proportion with the following insurance coverage of your clients with HIV in the past 12 months.

### 3.3 Pillar 1: Diagnosis

**Figure 1:**
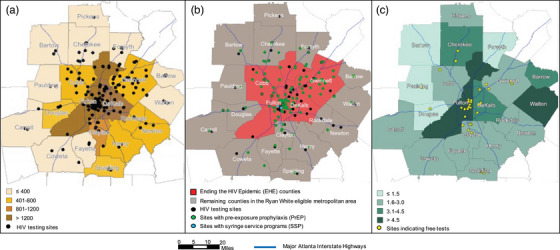
Atlanta eligible metropolitan area (EMA): (A) Rate of people living with HIV per 100,000 county population in 2021 and facilities with HIV testing (*N* = 177); (B) Ending the HIV Epidemic jurisdictions and sites with HIV testing, pre‐exposure prophylaxis (*N* = 130) and syringe service programmes (*N* = 2); (C) Rate of HIV testing sites per 100,000 county population and sites with free HIV tests (*N* = 37). Credits: Atlanta Regional Commission; Atlanta Regional Commission | GA Dept. of Transportation (GDOT), Atlanta Regional Commission | Ministry of Health, April 2019. HIV testing, PrEP and SSPs facilities obtained from CDC Get Tested; CDC CAPUS; and North America Syringe Exchange Network (NASEN) websites, respectively (data as of July 2022). Rate of PLHIV derived from AIDSVu database (2021 data): Sullivan PS, Woodyatt C, Koski C, Pembleton E, McGuinness P, Taussig J, Ricca A, Luisi N, Mokotoff E, Benbow N, Castel AD. A data visualization and dissemination resource to support HIV prevention and care at the local level: analysis and uses of the AIDSVu Public Data Resource. Journal of Medical Internet Research. 2020;22(10):e23173. Free indicates tests are provided at no‐cost regardless of insurance coverage.

We identified 177 total sites offering HIV testing in the EMA (Figure 1); 77 (43%) of these sites offered rapid HIV tests and 93% conventional laboratory tests. One hundred twenty‐three (70%) sites were located within the four EHE counties (Supplementary Table A2). Five non‐EHE counties with comparable rates of PLHIV to EHE counties (Clayton, Douglas, Henry, Newton and Rockdale) accounted for 16% of the EMA's new diagnoses, yet <9% of its testing sites. Overall, only 37 (21%) of all sites offered HIV tests at no cost (25 in EHE counties) and only six sites (five in EHE counties) offered HIV self‐testing kits.

In 2021, the DPH supported a total of 37,916 tests among EMA residents, 73% of which were among EHE jurisdiction residents (Supplementary Table A3), compared to 78% of the EMA's tests conducted at testing sites within EHE counties—meaning nearly 1800 tests conducted in EHE counties were among people residing in other parts of the EMA. Clayton, Douglas, Henry, Newton and Rockdale County residents accounted for 14% of the EMA's tests, but sites within these counties represented only 11% of the tests delivered.

Unmet need for HIV testing services ranged from 29% of respondents indicating a high unmet need for conventional HIV testing to 59% indicating a high unmet need for mobile clinics testing (Table 3). Despite that only 29% and 37% of respondents indicated HIV self‐testing kits and mobile clinic testing, respectively, were available at their organization, only a slight majority of respondents (50% and 59%, respectively) indicated there was high unmet need for these services. Nine respondents (21%) selected testing for scale‐up/implementation (ORIC ratings ranging from 42% of responses indicating readiness for implementing conventional HIV testing to 96% for HIV self‐testing kit provision).

**Table 3:** Stakeholder‐reported service availability within healthcare agencies and perceived unmet need by EHE Pillar and activity

Service	Performed by agency onsite (*N*; %)^a^	Percent of sites offering service (Median [Q1, Q3])^b^	Unmet need rating ≥ 4 (*N*; %)^c^
*Pillar 1: Diagnosis*			
Routine (opt‐out) HIV testing	35 (81)	100 (63, 100)	13 (42)
Conventional HIV testing (test sent to lab)	32 (76)	100 (−)	10 (29)
Rapid HIV testing (results 30 minutes onsite)	26 (62)	100 (63, 100)	16 (46)
HIV self‐testing kits provision	12 (29)	50 (60, 100)	17 (50)
Mobile clinics testing	16 (37)	100 (88, 100)	22 (59)
HIV test counselling	35 (83)	100 (80, 100)	12 (35)
*Pillar 2: Prevention*			
Pre‐exposure prophylaxis (PrEP) prescription	35 (81)	100 (20, 100)	13 (39)
Pre‐exposure prophylaxis (PrEP) navigation	37 (86)	100 (56, 100)	13 (39)
Pre‐exposure prophylaxis (PrEP) adherence counselling	33 (77)	75 (15, 100)	17 (49)
Non‐occupational post‐exposure prophylaxis (nPEP) prescription	24 (57)	50 (10, 100)	13 (41)
Non‐occupational post‐exposure prophylaxis (nPEP) navigation	24 (56)	30 (30, 100)	12 (38)
Condom distribution	31 (70)	100 (−)	11 (33)
Syringe service programmes	7 (16)	0 (50, 100)	14 (44)
*Pillars 3,4: Treatment, Response*			
HIV case management	33 (75)	100 (−)	18 (51)
HIV medication adherence education/counselling	32 (73)	100 (−)	18 (50)
HIV health appointment reminders (mobile/SMS)	22 (51)	100 (−)	16 (52)
Partner services: notification and counselling	20 (45)	100 (50, 100)	14 (38)
*Community and social support services*			
Peer outreach and care navigation	28 (64)	100 [−]	20 (59)
Translation (linguistic) services	29 (66)	100 [20, 100]	12 (33)
*Concurrent/integrated care*			
Mental healthcare	35 (83)	100 (60, 100)	20 (59)
Substance use care	28 (65)	20 (20, 100)	21 (58)
Telehealth appointments	32 (74)	100 (−)	8 (26)
STI testing	38 (88)	100 (55, 100)	14 (41)
STI treatment	36 (84)	100 (70, 100)	14 (40)
On‐site pharmacy	23 (53)	100 (50, 100)	9 (32)
Drug purchasing assistance programmes	21 (49)	50 (−)	12 (39)
*Services addressing social determinants of health*			
Housing services	26 (59)	100 (−)	22 (63)
Food and nutrition programmes	22 (50)	80 (58, 70)	18 (51)
Health promotion/education programmes	32 (73)	100 (−)	16 (47)
Transportation services to health appointments and pharmacies	29 (66)	100 (−)	23 (64)

^a^ Percentage from total responses.

^b^ (Applies for organizations with multiple locations) If service is provided onsite, value presented as percentage of all sites within the organization offering the service.

^c^ Unmet need rating: 5 = Highest unmet need; 4 = High unmet need; 3 = Neutral; 2 = Low unmet need; 1 = Lowest unmet need).

Stakeholders otherwise highlighted barriers in the implementation of opt‐out and routine HIV testing and the need to increase routine HIV/ sexually transmitted infections (STI) testing (Supplementary Table A4).

*“From my point of view, most practices don't engage in HIV testing efforts unless the patient asks for it or other sexually transmitted infection testing. I have noticed that within our Latinx community, we are quicker to test for type 2 diabetes (T2DM) (A1c, Accu‐check, lipid, and CMP panel), which is a warranted mindset given data on T2DM affecting our community.”—Community‐based organization employee*

*“We can definitely have a higher HIV test rate if there are more subsidized resources for testing or insurance coverage.”—Primary care physician*

### 3.4 Pillar 2: Prevention

We identified 130 PrEP sites in the EMA, >84% of which were located North of the Interstate 20 (I‐20) Highway, and only two sites for SSP (both in Fulton County). EHE counties represented 75% of the EMA's PrEP locations. The southern EMA counties with comparable rates of new diagnoses to EHE jurisdictions (Clayton, Douglas, Newton and Rockdale) accounted for 14% of the EMA's new HIV diagnoses yet <5% of its PrEP locations (Figure 1 and Supplementary Table A2).

In 2021, GDPH reported 31,877 PrEP eligibility screenings within the EMA, 81% were within EHE jurisdictions, while a lower proportion (76%) of PrEP screenings were among people residing in the EHE counties—meaning nearly 2000 screenings within EHE counties were among people residing within other EMA counties (Supplementary Table A3). Residents of Clayton, Douglas, Newton and Rockdale represented 12% of PrEP screenings, but sites within these counties accounted for 10% of EMA screenings. PrEP‐to‐Need ratios among several non‐EHE counties in the Atlanta EMA (e.g. Clayton: 2.53) reflected unmet need over three times higher relative to the EHE counties.

Perceived unmet needs for prevention services ranged from 33% of respondents indicating high unmet need for condom distribution to 49% indicating high unmet need for PrEP adherence counselling (Table 3). Syringe services were least available with only 16% of respondents reporting this offered, while PrEP navigation was the most commonly available (86% of respondents reported it available). Eleven respondents (26%) selected prevention services as most preferred for scale‐up/implementation (Supplementary Table A5). PrEP prescription scale‐up or implementation was among the most frequently selected, and 60% of responses for this intervention indicated organizational readiness for this change. ORIC ratings were lowest for PrEP navigation and SSPs (42% of responses agreement).

PrEP experience was surveyed among 24 HIV specialists and other prescribers (Supplementary Table A6). A lower proportion of non‐HIV specialist prescribers felt they were able to determine if PrEP was indicated for their patients (75%), were comfortable prescribing PrEP (63%) and had ever prescribed PrEP (63%) for HIV prevention, compared to HIV specialists (94%, 94% and 88%, respectively). Respondents highlighted structural barriers in the access, provision and continuum of care for PrEP, as well as the need for comprehensive HIV prevention services (Supplementary Table A4).

*“The number one choice would be PrEP treatment (being able to have the funded capacity to provide additional provider hours, lab coverage, for those who are UNINSURED). That is where the gap lies. People with insurance have access to PrEP treatment and the meds. The uninsured do not have equal access and as a result they tend to have the highest risk for HIV acquisition.”—Community‐based organization employee*

### 3.5 Pillar 3: Treatment

In 2021, GDPH reported that 62% of PLHIV in the EMA were virally suppressed. Viral suppression ranged from 59% (Fulton) to 66% (Gwinnett) in EHE jurisdictions and from 59% (Spalding) to 74% (Fayette) across other EMA counties (Supplementary Table A3).

Over 50% of survey respondents indicated high unmet needs for HIV case management, antiretroviral therapy (ART) adherence counselling and health appointment reminders (Table 3); however, only two respondents prioritized treatment services for scale‐up or implementation (54% of ORIC responses indicating readiness for HIV case management implementation) (Supplementary Table A5). Respondents highlighted the negative impacts of the COVID‐19 pandemic on HIV service delivery, and human resources for case management, navigation, treatment adherence and other clinical support (Supplementary Table A4).

*“COVID‐19 dismantled the sexual health and HIV services at [name redacted]* *. Additionally, the Boards of Health throughout Georgia became statewide entities last July 2021, and with that we lost 80% or more of our staff in the transition…”—Health agency employee*

*“For HIV case management, I would like didactics/collaboration from infectious disease on starting and maintaining ART.”—Primary care physician*

### 3.6 Pillar 4: Respond

Among CDC‐funded HIV testing and partner services, in 2021, only 54% of persons newly diagnosed with HIV in Georgia were interviewed for partner services (24% when including missing/invalid data), compared to 75% overall across CDC‐funded jurisdictions [34]. GDPH data indicate only 290 PLHIV newly diagnosed in the EMA were interviewed for partner services by the DPH in 2021 (only 17% of the EMA's new diagnoses). People residing within EHE counties made up 84% of the EMA's partner services interviews (Supplementary Table A3).

Only 45% of respondents indicated that partner services were offered by their agency and 62% felt the unmet need for this service was not high (Table 3). Only one respondent selected partner services for implementation in their organization; however, 67% of their responses indicated low organizational readiness for implementation (Supplementary Table A5). The limited availability of partner services was highlighted by respondents across care settings (Supplementary Table A4).

*“Partner services are available for veterans only.”—Infectious Disease Physician*

### 3.7 Integrated care, supportive services and policy

Most respondents indicated a high unmet need for peer outreach and care navigation (59%), mental healthcare (59%), substance use care (58%), housing services (63%), food and nutrition programmes (51%) and transportation services for health appointments and pharmacies (64%) (Table 3). Peer outreach, mental health services and housing services were the most commonly selected for scale‐up or implementation with organizational readiness ranging from 48% of responses for housing services to 71% for peer outreach and care navigation (Supplementary Table A5).

Respondents highlighted limited availability of mental health and substance use care, housing and transportation services, in addition to the need for meaningful engagement with people most affected by HIV, increased funding for peer support and Medicaid expansion (Supplementary Table A4).

*“Though we may provide most of the services listed, there are services that we just don't have enough capacity to serve all in need i.e. housing, mental health, transportation in rural areas.”—Community‐based organization employee*

*“…I've found that many members of this community are very isolated and suffer in silence, but they don't seek out or accept traditional mental health services. I think as HIV organizations we get caught up with meeting the ‘numbers’, and meeting ‘deliverables’ which sometimes causes us to miss genuine and authentic opportunities to engage with the people sitting right in front of us. It's a delicate balance. I think that funding that is less restrictive and allows for flexibility to meet clients where they are at is necessary.”—Community‐based organization employee*

### 3.8 Health system capacity and infrastructure

Thirteen respondents additionally completed the supplemental staffing questionnaire (Supplementary Table A7). The median number of staff working in HIV services across organizations was 14 (Q1,Q3:6,15) with a median of 2 (Q1,Q3:1,4) current vacant positions for HIV service staff. There were a median of 3 (Q1,Q3:1,4) peer staff working mainly in outreach, but also in care navigation/other roles. Organizations reported the capacity to hire an additional 1.5 FTE staff in peer care navigation roles. Median salaries ranged from a minimum of $35,000 and $36,000 for peer workers and epidemiologists/data analysts, respectively, to a maximum of $130,000 and $199,364 for nurse practitioners and public health physicians, respectively.

Respondents highlighted the need for anti‐stigma, anti‐racism, lesbian, gay, bisexual, transgender, queer‐(LGBTQ+), cultural awareness‐, sensitivity‐ and trauma‐informed care training, the need for community‐driven leadership in the EHE response, and the need for higher wages for health agency staff (Supplementary Table A4).

*“We need to improve our reputation with community. We also need to actively recruit for our vacant roles. In addition, we need to train staff so we create a more queer‐ and trans‐friendly environment. Paying for training, though impactful, diversity trainings are limited and I am not confident they work at shifting the work culture.”—Health agency employee*

*“We need to pay higher wages for the work we do. We can write this into our budget but the state system has caps and restrictions on what we can pay for these roles, and they are the same as other smaller counties with different costs of living.”—Health agency employee*

*“Create structure for leadership driven by community (including healthcare providers) for metro Atlanta's EHE Initiative; regular meetings among all stakeholders to provide updates, revise action plans, and evaluate outcomes.”—Infectious disease physician*

Georgia's EHE plan, released in 2020, requested $96M in funding over 5 years to support seven proposed goals and 26 activities across the EHE pillars—of which 95% was allocated to treatment services (Supplementary Figure A2 and Table A8).

## 4 DISCUSSION

This mixed‐methods study examining geographic HIV epidemiology and service data presents a novel approach to integrate surveys and implementation science methodology at a local level to determine community unmet needs and identify a range of key priorities to address for a metropolitan area's HIV/AIDS response. Our analysis highlighted racial/ethnic and geographic inequities in service access and disparities in HIV incidence. Several non‐EHE counties in the southern Atlanta EMA were particularly underserved in HIV testing and prevention services. Survey respondents indicated a high unmet need for self‐testing kits and mobile clinic testing, HIV case management, housing and transportation services as well as mental health and substance use care. Respondents otherwise indicated health agency staffing and infrastructure were currently insufficient to promote staff buy‐in and facilitate effective sustainment for most of the services in need of expansion across EHE pillars.

Filling vacancies in key leadership roles responsible for coordinating the overall HIV response in Atlanta, as well as staff shortages throughout the HIV response infrastructure are perhaps the primary and most immediate needs to address that could improve the local HIV/AIDS response. The role that has primary overall responsibility for coordinating the HIV response in Atlanta and throughout Georgia was vacant for 6 months at the time of writing. Staffing shortages due to low pay and pandemic burnout challenge all aspects of the public health workforce across the United States [44, 45, 46]. The EHE programme's current focus on only four of the EMA's 20 counties (based on 2017 HIV incidence), and the wide variation in public health infrastructure and resources between these counties pose significant challenges in effectively coordinating a cohesive regional strategy. Ongoing gentrification of central neighbourhoods [47] and disinvestment in clinical services for historically excluded, minoritized and underserved populations will exacerbate this challenge as poorer populations are displaced to counties where services are less accessible. Increased use of existing facilities and innovative delivery strategies, less tied to physical location (e.g. E‐health, telehealth, mobile health units and medication delivery) will be required to reach the scale necessary to meet EHE goals.

Expanding HIV testing is perhaps the highest priority that simulation modelling analyses have identified [48]. CDC initiatives offering free HIV self‐testing kits by mail have expanded nationally [49]; however, the limited number of sites offering free tests across the EMA remains a critical barrier despite free HIV testing being a noted priority of the Fulton County HIV Task Force. Despite CDC recommendations for routine opt‐out HIV testing in healthcare settings since 2006 [50], this is currently only implemented at Grady Memorial Hospital (co‐owned by Fulton and Dekalb counties) to our knowledge. Even if HIV tests were offered across all Atlanta primary care centres, strategies to optimize test offer, acceptance and subsequent linkage to care would need to accompany these changes. Finally, although HIV transmission through injection drug use is relatively low in Georgia [51], needle distribution services are inexpensive and highly effective in preventing HIV transmission and related outbreaks from injection drug use. These services remain constrained in Atlanta despite recent legal changes for SSP authorization and low‐threshold, community‐based programmes including mobile health units must be prioritized in HIV prevention efforts.

While only 29% of respondents indicated that HIV self‐testing kits were available at their organization, only 50% indicated unmet need for this service within their community. Several respondents indicated the need for more subsidized resources and insurance coverage to increase HIV testing—despite that insurance plans are required to cover testing without cost‐sharing [52], highlighting the importance for the promotion of testing and coverage. Additionally, less than 40% indicated an unmet need for partner services within their community, and while partner services are primarily the role of local and state health departments, only 17% of PLHIV newly diagnosed in 2021 were interviewed for partner services by the DPH—indicating higher unmet need for this service in the EMA than reported.

Similar to HIV testing sites, we found that <20% of PrEP sites in the EMA were located south of the I‐20 Highway (a historical boundary for residential segregation between Black and White communities in Atlanta [53]). PrEP is a highly effective intervention and a key component of the EHE initiative; however, its unequal access and uptake across racial/ethnic communities, particularly among Black men who have sex with men, has contributed to exacerbated racial disparities in HIV incidence [54]. Furthermore, recent reports that less than 5% of PrEP clients incur out‐of‐pocket costs nationwide [55] likely understate financial barriers posed by costs, instead reflecting how seldom individuals will use PrEP if they are required to pay. Limited awareness and misperceptions of risk [56] are also factors in this decision; however, lab and clinical costs can be additional barriers for uninsured individuals aware of PrEP. A call to consider Medicaid expansion and PrEP Drug Assistance Programs is furthermore warranted, given these policies are associated with more equitable PrEP use at the state level [57]. Our data also highlight PrEP prescribing gaps among non‐HIV specialists. PrEP expansion in primary care settings can be a key strategy to increase PrEP access through provider education alongside optimized protocols [58].

In 2021, Black/African Americans accounted for 21% of PrEP users in the South despite 52% of new HIV diagnoses in 2020 [59], highlighting an urgent need to identify policies and programmes to improve PrEP uptake and equity. In addition to expanding PrEP services to areas with inequitable access, interventions to increase PrEP uptake within Black and Latinx communities with high HIV incidence and low PrEP use must be developed and could involve resourcing community‐based organizations (particularly organizations led by Black and Latinx community members) to offer PrEP or connect those eligible to partner organizations providing PrEP.

Unlike HIV testing and PrEP, ART is available to uninsured and underinsured residents through the RWHAP. Nevertheless, relatively low rates of viral suppression indicate access and sustained retention in ART are persistent challenges throughout the EMA. RWHAP provides travel vouchers to offset the costs of travelling to clinics, but travel to pharmacies may also pose barriers, since only select pharmacies in the EMA dispense antiretrovirals to AIDS Drug Assistance Program (ADAP) clients. Washington State reported a 26% disenrollment rate among ADAP clients due to the 6‐month recertification criterion [60]. While federal policy requires a 6‐month ADAP recertification period, some states (including Georgia) allow eligibility renewal every 12 months [60]; these policies should be further modernized to reduce the administrative burden on clients and staff. Notably, various forms of case management have been used to promote ART retention with modest effectiveness; however, given the resource intensiveness and low ART retention rates in Atlanta, these interventions provided limited value without other interventions to increase ART uptake [24].

Finally, simple, low‐cost measures can be taken to combat HIV stigma and increase awareness of testing and prevention measures in communities that would most benefit. New York City's rebranding of all STI/STD clinics as sexual health clinics coincided with a 15% increase in usership [61]. Status‐neutral sexual health clinics represent a low‐cost intervention to reduce stigma—an example all settings, particularly Atlanta, should follow. However, this change alone is insufficient to address the persistent stigma associated with HIV. Thus, community‐designed and ‐led efforts must be developed and, importantly, supported by county, state and federal funders [62]. Given the lasting effects of segregation and persistent structural racism, community empowerment must form the foundation of the Atlanta HIV/AIDS response.

This study has several limitations. First, our respondents were not randomly selected and thus may not be a representative sample of experts working across all EMA counties. We note a small study sample size (particularly for the staffing supplement) and low response rate during recruitment in part due to the COVID‐19 pandemic and Mpox public health response and it is possible some perspectives were under‐represented. Nonetheless, we surveyed a majority of contacts referred from experts working within major HIV care agencies, including district public health departments ensuring all health districts in the EMA were represented. Second, GDPH‐reported testing estimates did not account for CDC‐funded self‐tests. Third, county‐level data for PrEP use stratified by race/ethnicity were unavailable in the databases, highlighting a priority metric urgently needed in health surveillance to identify and address racial inequities in PrEP access at the community level. Despite these challenges, this study offers comprehensive geographic data on the scope and availability of HIV services, organization infrastructure and climate for service expansion, as well as community recommendations to support the public health response, and can serve as a framework to evaluate HIV service provision in other settings.

## 5 CONCLUSIONS

Service delivery for each pillar of the EHE initiative must substantially expand to successfully reach national goals in metro Atlanta. Geographic disparities in HIV incidence and inequities in access to care persist in several counties in the south of the EMA which are impacted by their inclusion in RWHAP decisions but their exclusion from EHE resources. Increased federal and state investments in public health infrastructure, which we have previously estimated as providing immense public health value [24], can support local leadership and those working on the frontlines towards implementing equitable access to care.

## COMPETING INTERESTS

PSS reports grants and consulting fees from Gilead Sciences, Merck and Viiv Healthcare. GD reports grant funding from Gilead Sciences through the Gilead Research Scholars programme. CdR reports grants from NIH/NIAID [P30AI50409], and consultant payments from Resverlogics and Roche Diagnostics.

## AUTHORS’ CONTRIBUTIONS

BN, CdR and MP conceptualized the study. MP and BN wrote the first draft of the article. MP and BN executed the analysis and produced the tables and figures. JCS, BY, BE, XZ, PSS, WSA, MAT, GD and CdR aided in the interpretation of results and provided critical revisions to the article. BN secured funding for the study. All authors approved the final draft.

## FUNDING

The study was supported by the National Institutes of Health/National Institute on Drug Abuse grant no. R01‐DA041747 and a Fulbright Canada scholarship award.

## DISCLAIMER

The content is solely the responsibility of the authors and does not necessarily represent the official views of the funding agencies or data stewards.

## Supporting information

Additional file 1: “Supplementary Appendix A”
Contains supplementary information on study results.

Additional file 2: “Supplementary Appendix B”
Contains supplementary information on study results.

## Data Availability

The public data sources used are cited in the references and additional data that supports the findings of this study are available in the Supplementary Material of this article.
