# Supplementary material for: Ending the HIV Epidemic in Metropolitan Atlanta: a mixed‐methods study to support the local HIV/AIDS response
Source: J Int AIDS Soc. 2024 Jul 22;27(7):e26322. doi: 10.1002/jia2.26322 (PMC11263453; doi:10.1002/jia2.26322)
Supplement: Supplementary file 1 — Additional file 1: “Supplementary Appendix A” Contains supplementary information on study results. [file JIA2-27-e26322-s001.docx]

**SUPPLEMENTARY APPENDIX A**

**Ending the HIV Epidemic in Metropolitan Atlanta: A mixed-methods study to support the local HIV/AIDS response**

Micah Piske^1^, Bohdan Nosyk^1,2§^, Justin C Smith^3,4^, Bianca Yeung^1^, Benjamin Enns^1^, Xiao Zang ^5^, Patrick S Sullivan^6^, Wendy S Armstrong ^7,10^, Melanie A Thompson^8^, Gaea Daniel^9^, Carlos del Rio ^7,10^.

1. Centre for Advancing Health Outcomes, St. Paul’s Hospital, Vancouver, Canada;
2. Faculty of Health Sciences, Simon Fraser University, Burnaby, Canada;
3. Positive Impact Health Centers, Atlanta, USA;
4. Harvard T.H. Chan School of Public Health, Boston, USA
5. Division of Health Policy and Management, School of Public Health, University of Minnesota, Minneapolis, USA;
6. Department of Epidemiology, Emory University, Rollins School of Public Health, Atlanta, USA;
7. Division of Infectious Diseases, Department of Medicine, Emory University School of Medicine, Atlanta, USA;
8. Thacker & Thompson, MD, Atlanta, USA;
9. Nell Hodgson Woodruff School of Nursing, Emory University, Atlanta, USA;
10. Grady Health System, Atlanta, USA.

| **Contents** | |
| --- | --- |
| A1. Geographic boundaries included in study | 2 |
| Table A1. Description of survey components | 3-4 |
| Table A2. Description of HIV testing and Pre-exposure prophylaxis (PrEP) locations by county | 5 |
| Table A3. Georgia Department of Public Health obtained data for supported testing events, PrEP screenings, partner services interviews and viral suppression | 6 |
| Table A4. Key themes and representative quotations from qualitative analysis of survey responses | 7-8 |
| Table A5. Organizational readiness for implementing change (ORIC) assessment results | 9 |
| Table A6. PrEP experience among HIV specialists and other prescribers | 10 |
| Table A7. Organization infrastructure, staffing, vacancies, and salaries | 11 |
| Figure A2. Ending the HIV Epidemic (EHE) in Georgia, State EHE Plan (2020) budget allocations | 12 |
| Table A8. Ending the HIV Epidemic (EHE) in Georgia, State EHE Plan (2020) goals, activities, proposed funding allocation details | 13-14 |
| Appendix References | 15 |

**A1.0** **Boundaries included in study**

Ryan White Eligible Metropolitan Area (EMA) of Atlanta and rationale

**Figure A1.** Atlanta, GA EMA regional boundaries included in analysis (EHE jurisdictions indicated in red)


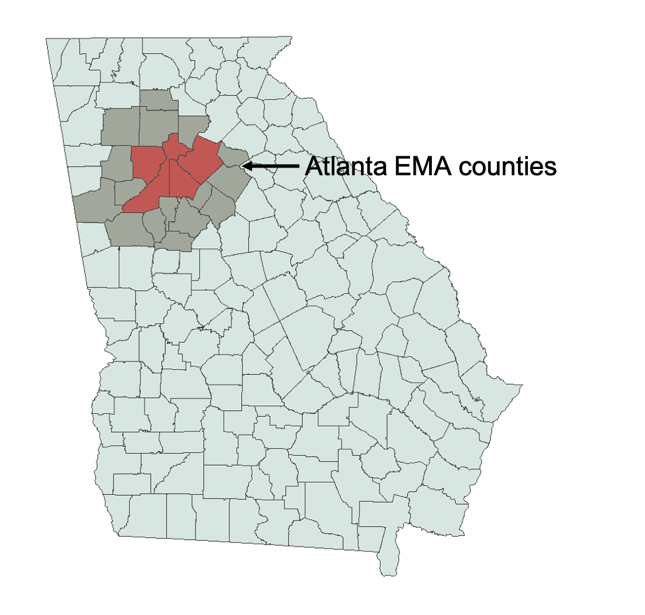


20 Counties were included in Atlanta: Barrow, Bartow, Carroll, Cherokee, Clayton, **Cobb**, Coweta, **DeKalb**, Douglas, Fayette, Forsyth, **Fulton**, **Gwinnett**, Henry, Newton, Paulding, Pickens, Rockdale, Spalding, and Walton. Counties included in city boundaries for Atlanta were determined from those included in the definition of the Ryan White HIV/AIDS Program Part A Eligible Metropolitan Area (EMA). The four Counties in bold (and highlighted in red in Figure A1) indicate those targeted by the ‘End the HIV Epidemic’ (EHE) initiative.

Relevant to these geographic boundaries, according to the Fulton County government, *“the purpose of the Atlanta Eligible Metropolitan Area (EMA) Ryan White Part A program is to improve the availability and quality of care for low-income, uninsured, and underinsured individuals and families affected by HIV in the 20-County Atlanta EMA (1). Funds support clients as they progress through the HIV care continuum and include core medical and support services. The program serves a large number of people living with HIV1 (PLHIV) living in poverty, afflicted with other medical conditions, and lacking health insurance (1). This includes a sizable population of PLHIV who are not eligible for insurance under the Affordable Care Act (ACA), or most publicly-funded programs (1). Ryan White Part A funds will be used to address service needs and gaps as the implementation of the ACA continues (1). Funds are also used to support the Quality Management (QM) program and Metropolitan Atlanta HIV Health Services Planning Council (Planning Council), the planning body that undertakes comprehensive planning activities, establishes service priorities and allocates funds in the EMA (1). Minority AIDS Initiative (MAI) funds are allocated to Outpatient/Ambulatory Health Services (OAHS) to improve health outcomes of people of color (1).”*

**Table A1.** Survey components, sources, description of content, respondents and number of items

| **Part** | **Survey component** | **Survey sources** | **Respondent** | **Description of content and measurements** | **Total No. of items** |
| --- | --- | --- | --- | --- | --- |
| I | Respondent demographics | N/A | All | Name, contact information, mailing address, organization/affiliation (if applicable), County location of affiliation (or County of residence if no affiliation), role/position, gender, race/ethnicity, (care providers: certifications, and expertise/ practice type) | 10 |
|  | Resource inventory/ availability and perceived unmet need | - Association of State and Territorial Health Officials (ASTHO) Profile Survey (2) format for availability of services adapted for HIV-related services, integrated care and services addressing social determinants of health - List of HIV and related services derived from CDC Compendium of Evidence-Based Interventions and Best Practices for HIV Prevention (3), CDC National Prevention Information Network (4), and County health department websites | All | - Services and activities provided directly on site by the health agency covering: HIV/AIDS prevention, testing, treatment and response (partner services), in addition to community-based service and supports, integrated care, and services that address social determinants of health. (non-agency staff/community members were asked whether they felt the service was available within the County they reside). Options: yes, no, or unsure; Additional component: estimated % of sites providing service directly if multi-site agency (Administrators/agency staff only) - Perceived unmet need by service according to the community served (5-point Likert scale: 5= Highest unmet need; 4= High unmet need; 3= Neutral; 2= Low unmet need; 1= Lowest unmet need) - Free text questions: 1. Please describe any relevant caveats or clarifications to your response regarding the available services above if needed; 2. What (if any) specific services and/or resources do you feel are still needed to address your community’s needs (if not covered previously)?; 3. If applicable, please describe how the services you indicated are delivered (including any relevant caveats to their availability or provision) if needed | 31 |
|  | Profile of client/patient demographics | Research literature and feedback from scientific advisory committee members/ other local stakeholders | All health agency staff | Number of patients or clients with HIV respondent has provided continuous and direct care or support for in the past 12 months, number of hours devoted to patient care or client support per week; demographics of patients or clients with HIV in the last 12 months: racial/ethnic makeup (%); HIV risk group makeup (%); insurance coverage level (%) | 5 |
|  | PrEP prescribing experience | Research literature including previous prescriber assessments (5), and feedback from scientific advisory committee members/ other local stakeholders | Physicians, Nurse Practitioners, Physician Assistants | Agreement on six statements related to prescribing PrEP assessed: I am familiar with current research on PrEP safety and efficacy; I am familiar with current guidelines on PrEP eligibility; I am comfortable discussing HIV risk factors and sexual health with my patients; I can determine if PrEP is indicated for my patients; I am comfortable prescribing PrEP to my patients; I have prescribed or currently prescribe PrEP for HIV prevention (5-point Likert scale: 5= Agree; 4= Somewhat agree; 3= Neither disagree nor agree; 2= Somewhat disagree; 1= Disagree) | 6 |
|  | Organizational readiness for implementing change | Organizational readiness for implementing change (ORIC questionnaire) (6), format adapted for hypothetical scale-up or implementation of service most preferred by respondent | All health agency staff | Agreement on 12 statements related to perceived organization implementation climate for hypothetical scale-up or implementation of service most needed/ selected by respondent: People who work here feel confident that the organization can get people invested in implementing this change; people who work here are committed to implementing this change; people who work here feel confident that they can keep track of progress in implementing this change; people who work here will do whatever it takes to implement this change; people who work here feel confident that the organization can support people as they adjust to this change; people who work here want to implement this change; people who work here feel confident that they can keep the momentum going in implementing this change; people who work here feel confident that they can handle the challenges that might arise in implementing this change; people who work here are determined to implement this change; people who work here feel confident that they can coordinate tasks so that implementation goes smoothly; people who work here are motivated to implement this change; people who work here feel confident that they can manage the politics of implementing this change (5-point Likert scale: 5= Agree; 4= Somewhat agree; 3= Neither disagree nor agree; 2= Somewhat disagree; 1= Disagree)   - Free text questions: 1. If you selected 'other (not initially listed)' please describe the intervention; 2.Please write in any relevant caveats or clarifications to your ratings above; 3. Please include details on any organizational barriers for implementing this change if applicable. | 12 |
| II | Optional: Agency staffing, infrastructure and salary ranges | Association of State and Territorial Health Officials (ASTHO) Profile Survey (2), Adapted to assess staff providing HIV-related services and peer outreach positions | All health agency staff with operations/ staffing knowledge | Estimated current number of staff members and full time equivalents providing HIV-related services, number of vacant positions for HIV-related positions, estimated number of peer navigators and outreach works, number of vacant positions for peer workers, total current FTE count and minimum to maximum annual salary ranges ($USD) by position: public health physician; public health nurse; nurse practitioner; peer worker; physician assistant; laboratory worker; behavioral health staff; epidemiologist/data analyst; health educator; public health informatics specialist; nutritionist; public information specialist; quality improvement specialist   - Free text questions: 1. Please write in any relevant caveats or clarifications to your response above if needed (optional); 2. What resources are needed to hire the necessary number of peer workers needed to serve your community? Please included details on funding if applicable. | 27 |

**Table A2**. Health care facilities in the Atlanta Eligible Metropolitan Area (EMA) with HIV testing and pre-exposure prophylaxis by testing type, cost, facility type, and distance from population center

| **County** | Rates of PLHIV (and new diagnoses), 2021 per 100,000^1^ | | No. PLHIV 2021(%) | | |  | | | No. HIV testing sites  (sites/  100,000 persons)^2^ | | | No. sites with PrEP  (sites/  100,000 persons)^2^ | | | **HIV test type & cost**  **(total, (%))** | | | | | | | | |  | | | | **HIV testing facility type**  **(total, (%))** | | | | | | | | | | |  | | | | **PrEP facility type**  **(total, (%))** | | | | | | | | | |  |  |  |  |  |  |  |
| --- | --- | --- | --- | --- | --- | --- | --- | --- | --- | --- | --- | --- | --- | --- | --- | --- | --- | --- | --- | --- | --- | --- | --- | --- | --- | --- | --- | --- | --- | --- | --- | --- | --- | --- | --- | --- | --- | --- | --- | --- | --- | --- | --- | --- | --- | --- | --- | --- | --- | --- | --- | --- | --- | --- | --- | --- | --- | --- | --- |
|  |  |  |  |  |  | No. new HIV diagnoses 2021  (%) | | |  |  |  |  |  |  | Sites with rapid tests | | | Sites with self-tests | | | Sites with free^3^ tests | | | | |  | | | | Clinic, FQHC, public health department^4^ | | Community-based organization | | | Pharmacy | | |  | | | | Clinic, FQHC, public health department^4^ | | | | Community-based organization | | | Pharmacy | | |  | | | |  |  |  |  |
| ***Ending the HIV Epidemic (EHE) counties*** | | | | | | | | | | | | | | | | | | | | | | | | | | | | | | | | | | | | | | | | | | | | | | | | | | | | | | | | | | |  |
| Cobb | | 565 (24) | | | 3,653 (9) | | | 152 (10) | | | 14 (1.8) | | | 13 (1.7) | | | 7 (50) | | | 0 | | | 1 (7) | | | |  | | | | 7 (50) | | | 1 (7) | | | 6 (43) | | | |  | | | | 6 (46) | | | 1 (8) | | 6 (46) | | | |  | | | |  |  |
| DeKalb | | 1,443 (54) | | | 9,140 (22) | | | 342 (22) | | | 28 (3.7) | | | 19 (2.5) | | | 13 (46) | | | 1 (4) | | | 4 (14) | | | |  | | | | 19 (68) | | | 5 (18) | | | 4 (14) | | | |  | | | | 13 (68) | | | 2 (10) | | 4 (21) | | | |  | | | |  |  |
| Fulton | | 1,802 (58) | | | 16,384 (40) | | | 525 (34) | | | 54 (5.1) | | | 40 (3.8) | | | 26 (48) | | | 3 (6) | | | 16 (30) | | | |  | | | | 41* (76) | | | 8 (15) | | | 5 (9) | | | |  | | | | 30 (75) | | | 6 (15) | | 4 (10) | | | |  | | | |  |  |
| Gwinnett | | 423 (21) | | | 3,347 (8) | | | 165 (11) | | | 27 (2.8) | | | 25 (2.6) | | | 9 (33) | | | 1 (4) | | | 4 (15) | | | |  | | | | 20 (74) | | | 2 (7) | | | 5 (19) | | | |  | | | | 18 (72) | | | 2 (8) | | 5 (20) | | | |  | | | |  |  |
| *Total EHE (%)* | | - | | | 32,524 (80) | | | 1,184 (77) | | | 123 (69) | | | 97 (75) | | | 55 (71) | | | 5 (83) | | | 25 (68) | | | |  | | | | 87 (69) | | | 16 (94) | | | 20 (61) | | | |  | | | | 67 (76) | | | 11 (100) | | 19 (61) | | | |  | | | |  |  |
| ***Remaining counties in Ryan White eligible metropolitan area (EMA)*** | | | | | | | | | | | | | | | | | | | | | | | | | | | | | | | | | | | | | | | | | | | | | | | | | | | | | | | | | | | |
| Barrow | | 194 (11) | | 138 (<1) | | | 8 (1) | | | 3 (3.5) | | | 2 (2.3) | | | 0 | | | 0 | | | 0 | | |  | | | | 3 (100) | | | | 0 | | | 0 | | | |  | | | | 2 (100) | | | 0 | | | | 0 | | | |  | |  |  |  |
| Bartow | | 249 (13) | | 231 (1) | | | 12 (1) | | | 2 (1.8) | | | 1 (0.9) | | | 0 | | | 0 | | | 0 | | |  | | | | 2 (100) | | | | 0 | | | 0 | | | |  | | | | 1 (100) | | | 0 | | | | 0 | | | |  | |  |  |  |
| Carroll | | 274 (11) | | 279 (1) | | | 11 (1) | | | 3 (1.7) | | | 3 (1.7) | | | 3 (100) | | | 0 | | | 0 | | |  | | | | 2 (67) | | | | 0 | | | 1 (33) | | | |  | | | | 2 (67) | | | 0 | | | | 1 (33) | | | |  | |  |  |  |
| Cherokee | | 195 (5) | | 452 (1) | | | 11 (1) | | | 9 (3.3) | | | 7 (2.5) | | | 3 (33) | | | 0 | | | 3 (33) | | |  | | | | 6 (67) | | | | 0 | | | 3 (33) | | | |  | | | | 4 (57) | | | 0 | | | | 3 (43) | | | |  | |  |  |  |
| Clayton | | **1049 (48)** | | 2,519 (6) | | | 115 (8) | | | 6 (2.0) | | | 4 (1.3) | | | 3 (50) | | | 1 (17) | | | 5 (83) | | |  | | | | 5 (83) | | | | 1 (17) | | | 0 | | | |  | | | | 4 (100) | | | 0 | | | | 0 | | | |  | |  |  |  |
| Coweta | | 242 (12) | | 305 (1) | | | 15 (1) | | | 3 (2.0) | | | 2 (1.3) | | | 2 (66) | | | 0 | | | 0 | | |  | | | | 2 (67) | | | | 0 | | | 1 (33) | | | |  | | | | 1 (50) | | | 0 | | | | 1 (50) | | | |  | |  |  |  |
| Douglas | | **595 (30)** | | 718 (2) | | | 36 (2) | | | 2 (1.4) | | | 1 (0.7) | | | 1 (50) | | | 0 | | | 1 (50) | | |  | | | | 1 (50) | | | | 0 | | | 1 (50) | | | |  | | | | 0 | | | 0 | | | | 1 (100) | | | |  | |  |  |  |
| Fayette | | 237 (-) | | 244 (1) | | | - | | | 2 (1.7) | | | 2 (1.7) | | | 0 | | | 0 | | | 0 | | |  | | | | 1 (50) | | | | 0 | | | 1 (50) | | | |  | | | | 1 (50) | | | 0 | | | | 1 (50) | | | |  | |  |  |  |
| Forsyth | | 191 (5) | | 196 (0.5) | | | 10 (1) | | | 4 (1.5) | | | 3 (1.2) | | | 2 (50) | | | 0 | | | 0 | | |  | | | | 3 (75) | | | | 0 | | | 1 (25) | | | |  | | | | 2 (67) | | | 0 | | | | 1 (33) | | | |  | |  |  |  |
| Henry | | **527 (17)** | | 1,076 (3) | | | 34 (2) | | | 4 (1.6) | | | 3 (1.2) | | | 1 (25) | | | 0 | | | 0 | | |  | | | | 2 (50) | | | | 0 | | | 2 (50) | | | |  | | | | 1 (33) | | | 0 | | | | 2 (66) | | | |  | |  |  |  |
| Newton | | **454 (22)** | | 692 (1) | | | 21 (1) | | | 3 (2.6) | | | 1 (0.9) | | | 1 (33) | | | 0 | | | 0 | | |  | | | | 2 (67) | | | | 0 | | | 1 (33) | | | |  | | | | 0 | | | 0 | | | | 1 (100) | | | |  | |  |  |  |
| Paulding | | 201 (14) | | 288 (1) | | | 20 (1) | | | 2 (1.2) | | | 1 (0.6) | | | 1 (50) | | | 0 | | | 1 (50) | | |  | | | | 1 (50) | | | | 0 | | | 1 (50) | | | |  | | | | 0 | | | 0 | | | | 1 (100) | | | |  | |  |  |  |
| Pickens | | 121 (-) | | 36 (<1) | | | - | | | 1 (2.9) | | | 0 - | | | 1 (100) | | | 0 | | | 0 | | |  | | | | 1 (100) | | | | 0 | | | 0 | | | |  | | | | 0 | | | 0 | | | | 0 | | | |  | |  |  |  |
| Rockdale | | **589 (46)** | | 465 (1) | | | 36 (2) | | | 2 (2.1) | | | 0 - | | | 1 (50) | | | 0 | | | 0 | | |  | | | | 2 (100) | | | | 0 | | | 0 | | | |  | | | | 0 | | | 0 | | | | 0 | | | |  | |  |  |  |
| Spalding | | 402 (11) | | 228 (1) | | | 6 (<1) | | | 2 (2.9) | | | 1 (1.5) | | | 1 (50) | | | 0 | | | 2 (100) | | |  | | | | 2 (100) | | | | 0 | | | 0 | | | |  | | | | 1 (100) | | | 0 | | | | 0 | | | |  | |  |  |  |
| Walton | | 338 (11) | | 282 (1) | | | 9 (1) | | | 6 (6.0) | | | 2 (2.0) | | | 2 (33) | | | 0 | | | 0 | | |  | | | | 5 (83) | | | | 0 | | | 1 (17) | | | |  | | | | 2 (100) | | | 0 | | | | 0 | | | |  | |  |  |  |
| *Total non-EHE (%)* | | - | | 8,149 (19) | | | 344 (23) | | | 54 (31) | | | 33 (25) | | | 22 (29) | | | 1 (17) | | | 12 (32) | | |  | | | | 40 (31) | | | | 1 (6) | | | 13 (39) | | | |  | | | | 21 (24) | | | 0 | | | | 12 (39) | | | |  | |  |  |  |
| **Total** | | - | | 40,673 | | | 1,528 | | | 177 | | | 130 | | | 77 | | | 6 | | | 37 | | |  | | | | 127 | | | | 17 | | | 33 | | | |  | | | | 88 | | | 11 | | | | 31 | | | |  | |  |  |  |

PLHIV: People living with HIV. Non-EHE jurisdiction counties with rate of PLHIV ≥423 (rate of EHE counties) indicated in bold.^1^ Rate of PLHIV from AIDSVu database (2021 data): Sullivan PS, Woodyatt C, Koski C, Pembleton E, McGuinness P, Taussig J, Ricca A, Luisi N, Mokotoff E, Benbow N, Castel AD. A data visualization and dissemination resource to support HIV prevention and care at the local level: analysis and uses of the AIDSVu Public Data Resource. Journal of medical Internet research. 2020;22(10):e23173; ^2^ Calculated by population estimates from US Census Bureau as of July 1, 2021. Facilities, type of HIV tests and costs from CDC Get Tested website (as of July 2022); ^3^ Free indicates tests are provided at no-cost regardless of insurance coverage; ^4^ Primary function is clinic, includes private clinics and social service organizations. *Includes n=3 sites classified as clinic/hospital.

**Table A3.** Georgia Department of Public Health-supported HIV **t**esting events and PrEP screening, viral suppression among people living with HIV and number of people newly diagnosed with HIV interviewed for partner services in the Atlanta Eligible Metropolitan Area (January 1, 2021 – December 31, 2021)

| **County** | **Pilar 1: Prevent** | |  | **Pilar 2: Diagnose** | |  | **Pilar 3: Treat** |  | **Pilar 4: Respond** |
| --- | --- | --- | --- | --- | --- | --- | --- | --- | --- |
|  | No. screened for PrEP eligibility* by client county  (%) | No. screened for PrEP eligibility* by site county |  | No. test events by client county (%) | No. test events by testing site county  (%) |  | PLHIV virally suppressed  (%) |  | HIV cases interviewed for partner services by client county |
| Barrow | 106 (0.3) | 36 (0.1) |  | 256 (0.7) | 173 (0.5) |  | 90 (64.3) |  | <5 |
| Bartow | 524 (1.7) | 526 (1.7) |  | 686 (1.8) | 553 (1.5) |  | 156 (67.2) |  | Undisclosed |
| Carroll | 464 (1.5) | 422 (1.3) |  | 518 (1.4) | 439 (1.2) |  | 166 (67.2) |  | <5 |
| Cherokee | 490 (1.6) | 476 (1.5) |  | 1,099 (2.9) | 1,004 (2.7) |  | 281 (68.9) |  | Undisclosed |
| Clayton | 1,665 (5.3) | 1,527 (4.8) |  | 1,910 (5.0) | 1,590 (4.2) |  | 1,838 (60.6) |  | 9 |
| Cobb | 3,769 (12.1) | 3,701 (11.6) |  | 4,473 (11.8) | 4,043 (10.7) |  | 2,313 (63.9) |  | 33 |
| Coweta | 248 (0.8) | 198 (0.6) |  | 514 (1.4) | 456 (1.2) |  | 194 (67.4) |  | <5 |
| DeKalb | 6,033 (19.4) | 7,354 (23.1) |  | 7,851 (20.7) | 9089 (24) |  | 6,000 (61.7) |  | 79 |
| Douglas | 394 (1.3) | 130 (0.4) |  | 695 (1.8) | 428 (1.1) |  | 468 (65.2) |  | <5 |
| Fayette | 167 (0.5) | 118 (0.4) |  | 303 (0.8) | 384 (1.0) |  | 176 (74) |  | Undisclosed |
| Forsyth | 79 (0.3) | 26 (0.1) |  | 147 (0.4) | 69 (0.2) |  | 164 (68) |  | <5 |
| Fulton | 10,418 (33.5) | 11,942 (37.5) |  | 11,644 (30.7) | 13,590 (36) |  | 9,509 (58.8) |  | 77 |
| Gwinnett | 3,487 (11.2) | 2,704 (8.5) |  | 3,837 (10.1) | 2,878 (7.6) |  | 2,408 (65.5) |  | 56 |
| Henry | 874 (2.8) | 510 (1.6) |  | 1,051 (2.8) | 536 (1.4) |  | 670 (66.4) |  | 6 |
| Newton | 930 (3.0) | 563 (1.8) |  | 974 (2.6) | 640 (1.7) |  | 264 (63.5) |  | 7 |
| Paulding | 209 (0.7) | 130 (0.4) |  | 329 (0.9) | 160 (0.4) |  | 273 (71) |  | <5 |
| Pickens | 112 (0.4) | 102 (0.3) |  | 158 (0.4) | 105 (0.3) |  | 27 (65.9) |  | Undisclosed |
| Rockdale | 721(2.3) | 1045 (3.3) |  | 763 (2.0) | 1,061 (2.8) |  | 282 (62.7) |  | 6 |
| Spalding | 280 (0.9) | 295 (0.9) |  | 514 (1.4) | 582 (1.5) |  | 134 (59.3) |  | <5 |
| Walton | 162 (0.5) | 72 (0.2) |  | 194 (0.5) | 89 (0.2) |  | 149 (66.8) |  | <5 |
| *# EHE (%)* | 23,707 (76) | 25,701 (81) |  | 27,805 (73) | 29,600 (78) |  | 58.8-65.5 |  | - |
| *# Non-EHE* | 7,425 (24) | 6,176 (19) |  | 10,111 (27) | 8,269 (22) |  | 59.3-74.3 |  | - |
| **Total EMA** | 31,132 | 31,877 |  | 37,916 | 37,869 |  | 25,561 (61.6) |  | 290 |

EHE: Ending the HIV Epidemic (jurisdictions in Georgia); EMA: Eligible metropolitan area; * Among persons testing negative for HIV.

**Table A4.** Key themes and quotations on reported resource needs and barriers in implementing services for HIV, integrated care, and social support programs by EHE Pillar and activity

| **Theme, Activity** | **Representative Quotations** | |
| --- | --- | --- |
| ***Pillar 1: Diagnosis*** | | |
| Implementation barriers in opt- out/routine HIV testing | | “From my point of view, most practices don't engage in HIV testing efforts unless the patient asks for it other STD testing. I have noticed that within our LatinX community, we are quicker to test for T2DM (A1c, Accu-check, lipid, and CMP panel), which is a warranted mindset given data on T2DM affecting our community.” - Community-based organization staff member |
| Need for improved insurance coverage for routine HIV/STI testing | | “We can definitely have a higher hiv test rate if there are more subsidized resources for testing or insurance coverage.” - Primary care physician |
| ***Pillar 2: Prevention*** | | |
| Limited availability of PrEP providers | “If we want to see a permanent lasting impact of PrEP, we have to have a sustainable infrastructure of resources to ensure that everyone has equal and easy access to PrEP care and treatment. Just like HIV care, people on PrEP that have been adherent, STAY on PrEP. Though it wasn't intended to be a lifetime treatment option, it has become that for most. So for the uninsured, this becomes an inaccessible, unsustainable, unpredictable option.”  - Community-based organization employee | |
| Insurance and inequitable access to sustainable PrEP | The number one choice would be PrEP treatment (being able to have the funded capacity to provide additional provider hours, lab coverage, for those who are UNINSURED). That is where the gap lies. People with insurance have access to PrEP treatment and the meds. The uninsured do not have equal access and as a result they tend to have the highest risk for HIV acquisition.  - Community-based organization employee | |
| Implement comprehensive HIV prevention | “This idea of taking comprehensive services to community is somewhat new and novel for most folks at [name redacted]. Folks internally are hyper focused on just HIV testing and don’t yet see the connection with comprehensive services and HIV prevention.” - Health agency employee | |
| ***Pillars 3,4: Treatment, Response*** | | |
| Impacts of COVID-19 pandemic on service delivery | “COVID-19 dismantled the sexual health and HIV services at [name redacted]. Additionally, the Boards of Health throughout Georgia became statewide entities last July 2021, and with that we lost 80% or more of staff in the transition. These results should change considerably in the next year, and they may have been better pre-COVID.” - Health agency employee | |
| Increase capacity and funding for case management, clinical navigation and treatment adherence | “We currently have one staff member (HIV Navigator) focusing on HIV and education efforts. However, given that this position is being funded via grants and is only temporary until December of this year. After grant funding expires, we will not have this service available until we get more funding, given we are a non-profit.” - Community-based organization employee | |
| Limited availability of partner services | “Partner services are available for veterans only.”  - Infectious Disease Physician | |
| Support and education for care providers and non-HIV specialists | “Telehealth expert consultation hotline for care providers with little or no expertise in HIV medicine as well as provider, agency, and community education (NOT from pharma) on long-acting injectables for PrEP and treatment.” - Infectious disease physician | |
| ***Integrated care & social support programs*** | | |
| Limited capacity and high demand for integrated care and social support | "Though we may provide most of the services listed, there are services that we just don't have enough capacity to serve all in need i.e. housing, mental health, transportation in rural areas." - Community-based organization employee | |
| Limited availability of health care for mental health and substance use | “We do not have a syringe program and we need to design and implement one in our District.” - Health agency employee  “Our organization provides MAT (suboxone) for people with OUD but they do not have counseling/mental health services for people with SUD” – Infectious disease physician | |
| Meaningful engagement and meeting clients where they are at | “I do believe that there's a need for deeper community engagement for the communities most affected by HIV/AIDS, beyond just outreach testing. I've found that many members of this community are very isolated and suffer in silence, but they don't seek out or accept traditional mental health services. I think as HIV organizations we get caught up with meeting the "numbers", and meeting "deliverables" which sometimes causes us to miss genuine and authentic opportunities to engage with the people sitting right in front of us. It’s a delicate balance. I think that funding that is less restrictive and allows for flexibility to meet clients where they are at is necessary.” - Community-based organization employee | |
| Sustained funding for community-based organization activities | “We need more funding to employ peer support staff. I think that there should be like a mobile mental health team that could help agencies such as AHA, Partners for home, Aniz Inc, Pads and other to help better assist the clients."  - Community-based organization employee | |
| Need for expanded housing and transportation services | “We need better housing supports, transportation and Medicaid expansion. Many housing services are only available for people living with HIV.” – Community advocate/person living with HIV | |
| ***Structural & Policy interventions*** | | |
| Limited drug assistance coverage and community involvement in decision-making | “ADAP is extremely slow to add new medications for PLHIV. Minimum participation to no participation of PLHIV to inform the process.”  - Community advocate/person living with HIV | |
| Anti-stigma, anti-racism training, LGBTQ and cultural awareness and sensitivity training and trauma-informed care | “We need to improve our reputation with community. We also need to actively recruit for our vacant roles. In addition, we need to train staff so we create a more queer- and trans-friendly environment. Paying for training, though impactful diversity trainings are limited and I am not confident they work at shifting the work culture.” - Health agency employee | |
| Ensure adequate living wages for care providers and public health staff | “We need to pay higher wages for the work we do. We can write this into our budget but the state system has caps and restrictions on what we can pay for these roles, and they are the same as other smaller counties with different costs of living.”  - Health agency employee | |
| Community-driven leadership for the local EHE response | “Create structure for leadership driven by community (including healthcare providers) for metro Atlanta's EHE Initiative; regular meetings among all stakeholders to provide updates, revise action plans, and evaluate outcomes.”  - Infectious disease physician | |
| Medicaid expansion | “I think if Georgia made a simple decision like expanding Medicaid access, we would see the health outcomes of people living with HIV improve significantly in this state. Healthcare access is a right that everyone should be afforded.”  - Community-based organization employee | |

Respondents were asked: 1. What (if any) specific services and/or resources do they feel are still needed to address their community’s needs (if not previously covered) and to 2. Include details on any organizational barriers for implementing or scaling up services to address unmet needs in their community.

**Table A5.** Interventions selected as most preferred for scale-up or implementation and perceived implementation climate (Organizational Readiness to Implement Change) (N=43)

|  |  |  |  | **ORIC Rating ≥ 4**  **N (%)^1^** | | | | | | | | | | | | |
| --- | --- | --- | --- | --- | --- | --- | --- | --- | --- | --- | --- | --- | --- | --- | --- | --- |
|  |  |  |  | **Change commitment** | | | |  | | | **Change efficacy** | | | |  | |
| **Service** | | Should be scaled up  N (%)^2^ | Should be implemented  N (%)^2^ | People who work here want to implement this change | We can get people invested in implementing this change | We are determined to implement this change | | |  | | We can manage the politics of implementing this change | | We can support people as they adjust to this change | We can coordinate tasks so that implementation goes smoothly | **Total**  **ORIC ratings^3^**  **≥ 4**  **(N, %)** | |
| ***Pillar 1: Diagnosis*** | | | | | | | | | | | | | | | | |
| Routine (opt-out) HIV testing | | - | 2 (100) | 1 (50) | 1 (50) | 1 (50) | | |  | | 1 (50) | | 2 (100) | 1 (50) | 14 (58) | |
| Conventional HIV testing (test sent to lab) | | - | 1 (100) | 0 | 1 (100) | 0 | | |  | | 0 | | 1 (100) | 0 | 5 (42) | |
| Rapid HIV testing (results 30 minutes on-site) | | - | 2 (100) | 2 (100) | 2 (100) | 0 | | |  | | 1 (50) | | 2 (100) | 1 (50) | 13 (54) | |
| HIV self-testing kits provision | | - | 2 (100) | 2 (100) | 2 (100) | 2 (100) | | |  | | 1 (50) | | 2 (100) | 2 (100) | 23 (96) | |
| Mobile clinics testing | | 2 (100) | - | 1 (50) | 2 (100) | 1 (50) | | |  | | 2 (100) | | 1 (50) | 1 (50) | 18 (75) | |
| ***Pillar 2: Prevention*** | | | | | | | | | | | | | | | | |
| Pre-exposure prophylaxis (PrEP) prescription | | **3 (75)** | **1 (25)** | 3 (75) | 3 (75) | 2 (50) | | |  | | 2 (50) | | 2 (50) | 2 (50) | 48 (60) | |
| Pre-exposure prophylaxis (PrEP) navigation | | - | 1 (100) | 1 | 0 | 1 | | |  | | 0 | | 0 | 1 | 5 (42) | |
| Non-occupational post-exposure prophylaxis (nPEP) prescription |  | - | 1 (100) | 1 | 1 | 1 | | |  | | 1 | | 1 | 1 | 12 (100) | |
| Non-occupational post-exposure prophylaxis (nPEP) navigation |  | - | 2 (100) | 2 (100) | 2 (100) | 1 (50) | | |  | | 2 (100) | | 2 (100) | 2 (100) | 12 (100) | |
| Syringe service programs |  | 2 (66) | 1 (33) | 1 (33) | 1 (33) | 1 (33) | | |  | | 1 (33) | | 2 (66) | 1 (33) | 15 (42) | |
| ***Pillars 3,4: Treatment, Response*** | | | | | | | | | | | | | | | | |
| HIV case management | | - | 2 (100) | 2 (100) | 0 | 1 (50) | | |  | | 0 | | 1 (50) | 1 (50) | 13 (54) | |
| Partner services: notification and counselling | |  | 1 (100) | 1 (100) | 0 | 0 | | |  | | 0 | | 0 | 1 (50) | 4 (33) | |
| ***Community and social support services*** | | | | | | | | | | | | | | | | |
| Peer outreach and care navigation | | **3 (75)** | **1 (25)** | 4 (100) | 4 (100) | 4 (100) | | |  | | 4 (100) | | 3 (75) | 4 (100) | 34 (71) | |
| Translation (linguistic) services | | - | 1 (50) | 1 | 1 | 1 | | |  | | 1 | | 1 | 1 | 12 (100) | |
| ***Concurrent/integrated care*** | | | | | | | | | | | | | | | | |
| Mental health care services | | **2 (50)** | **2 (50)** | 3 (75) | 3 (75) | 2 (50) | | |  | | 2 (50) | | 3 (75) | 2 (50) | 30 (63) | |
| Substance use services | | - | 1 (100) | 1 | 1 | 0 | | |  | | 1 | | 1 | 1 | 10 (83) | |
| Telehealth appointments | | - | 1 (100) | 0 | 0 | 0 | | |  | | 0 | | 0 | 0 | 0 | |
| STI testing | | 1 (100) | - | 0 | 1 | 0 | | |  | | 1 | | 0 | 0 | 6 (50) | |
| On-site pharmacy | | - | 1 (100) |  |  |  | | |  | |  | |  |  |  | |
| Drug purchasing assistance programs | |  | 1 (100) | 1 | 1 | 1 | | |  | | 1 | | 1 | 1 | 12 (100) | |
| ***Services addressing social determinants of health*** | | | | | | | | | | | | | | | | |
| Housing services | | **4 (75)** | **1 (100)** | 4 (75) | 2 (20) | 4 (75) |  | | | 1 (20) | | 1 (20) | | 1 (100) | | 29 (48) |
| Transportation services to health appointments and pharmacies | | 1 (100) | - | 1 | 1 | 1 | 1 | | | 1 | | 1 | | 1 | | 12 (100) |
| ^1^ 1=Disagree; 2= Somewhat Disagree; 3= Neither Agree nor Disagree; 4= Somewhat Agree; 5= Agree; ^2^ Percentage presented with total number of responses selecting this intervention as the denominator; ^3^ Total ratings from all 12 ORIC indicators including those not presented in the table. Respondents were asked: “From the list of services included which specific service or resource that is delivered (or could be delivered) by your agency would you m**ost** prefer to see implemented OR scaled-up/expanded in order to address unmet needs in your community?” For the second part of this question, respondents were asked: “Given the existing staffing infrastructure and capacity of your agency, to what extent would your agency or organization be able to implement or expand the service you identified in the previous question?” Services selected with four or more responses indicated in bold. | | | | | | | | | | | | | | | | |

**Table A6.** PrEP experience among HIV specialists and other prescribers (N= 24)

| **PrEP prescribing experience** | **Rating ≥ 4 (N;%)^1^** | | | | | |  |
| --- | --- | --- | --- | --- | --- | --- | --- |
|  | **Total** | | **HIV-Specialists^2^** | | **Other Prescribers^3^** | |  |
| "I am familiar with current research on PrEP safety and efficacy" | | 21 (88) | | 16 (100) | | 5 (63) | |
| "I am familiar with current guidelines on PrEP eligibility" | | 21 (88) | | 16 (100) | | 5 (63) | |
| "I am comfortable discussing HIV risk factors and sexual health with my patients" | | 23 (96) | | 16 (100) | | 7 (88) | |
| "I can determine if PrEP is indicated for my patients" | | 21 (88) | | 15 (94) | | 6 (75) | |
| "I am comfortable prescribing PrEP to my patients" | | 20 (83) | | 15 (94) | | 5 (63) | |
| "I have prescribed or currently prescribe PrEP for HIV prevention" | | 19 (79) | | 14 (88) | | 5 (63) |  |
| ^1^ (1=Disagree; 2= Somewhat disagree; 3= Neither disagree nor agree; 4= Somewhat agree; 5= Agree)  ^2^ Includes Infectious disease board-certified physicians, HIV specialists (HIVMA criteria, AAHIV-S, or other prescribers with HIV expertise) (N=16)  ^3^ Includes other board-certified physicians (family/internal medicine or other) and other prescribers (N=8) | | | | | | | |

**Table A7.** Organization infrastructure, salaries, and perceived resources required for staffing (N= 13 responses on staffing supplement questionnaire)

| **Organization capacity** | **Median (IQR)** |
| --- | --- |
| ***Current staff in HIV services*** | |
| Number of staff in HIV services | 14 (6, 15) |
| Number of Full-time equivalents (FTEs) | 10 (4, 14) |
| Number of part time workers | 1 (1, 3) |
| Number of hourly, casual or temporary staff | 1 (0, 3) |
| Total vacant positions for HIV services | 2 (1, 4) |
| ***Current staff in Peer outreach and care navigation*** | |
| Number of peer staff members | 3 (1, 4) |
| Number of peer Full-time equivalents (FTEs) | 2 (1, 2) |
| Number of peers in outreach | 3 (2, 4) |
| Number of peers in care navigation | 1 (1, 2) |
| Number of peers in other roles | 2 (1, 2) |
| ***Capacity for hiring additional peer staff*** | |
| Peer outreach FTEs | 1 (0.8, 1.3) |
| Peer care navigation FTEs | 1.5 (1.3, 1.8) |
| Other peer roles | 0 (0, 0.5) |
| **Occupation Salaries** | **Minimum, Maximum**  **(Median, $ USD)** |
| Public Health Physician | $155,708, $199,364 |
| Public Health Nurse | $50,634, $71,735 |
| Nurse Practitioner | $110,000, $130,000 |
| Peer worker | $35,000, $55,000 |
| Physician Assistant | $110,000, $130,000 |
| Laboratory Technician | $50,000, $80,000 |
| Behavioral Health Staff | $46,124, $61,342 |
| Epidemiologist/Data Analyst | $36,286, $63,469 |
| Health Educator | $54,987, $81,227 |
| Nutritionist | $49,987, $71,227 |

Respondents were asked the following questions by each section: 1. Please estimate the current number of staff members (include temporary and contract workers) and FTEs (including partial FTEs) working in your facility providing services within HIV prevention, testing, and treatment in the following categories (For example, a full-time employee is counted as 1.00 FTE. An employee who works part-time at 50% of the normal work hours for the position would be counted as a .50 FTE.) if you're unsure, please write in 'unsure'; 2. Peer outreach and navigation: If applicable at your facility, how many peer navigators or outreach workers does your facility currently employ? (if none enter '0'; if unsure, write 'unsure'). 'Peer' definition: a peer is someone living with HIV who contributes to positive health outcomes of other community members, but is not usually a health care professional with clinical training.; 3. For each occupational classification listed in the following table, please provide the annual salary range for staff working in your health agency/community organization

**Figure A2**. Ending the HIV Epidemic (EHE) in Georgia, State EHE Plan total requested 5-year budget allocations (2020), by Pillar (P), Goal (G) and Activity (A)


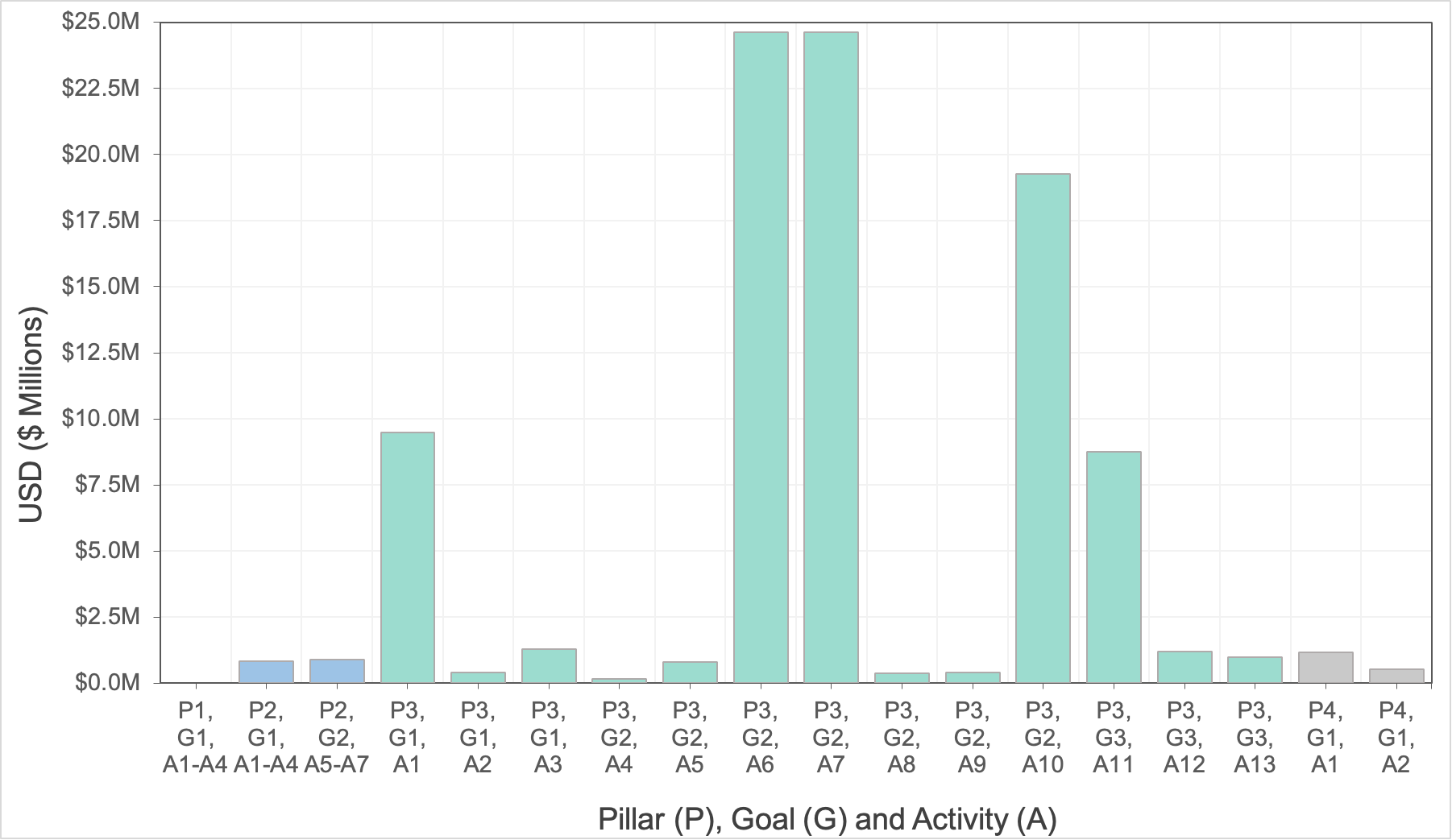


Allocations in figure and supporting table illustrated from the Ending the HIV Epidemic in Georgia Centers for Disease Control and Prevention EHE Plan; PS 19-1906 (December 2020). Available at: <https://www.cdc.gov/hiv/funding/announcements/ps19-1906/ehe-plans.html> [Accessed June 2, 2022]. Some activities without $ amounts defined were noted as ‘TBD’ for funding allocation estimates in the EHE plan (see corresponding table A2 on next page for further detail).

**Table A8**. Ending the HIV Epidemic (EHE) in Georgia, State EHE Plan (2020): Goals, activities, proposed funding resources and 5-year funding allocation details

| **Pillar, Goal** | | **Activity** | **Potential funding resources** | | **Total requested funding allocation** | |  |
| --- | --- | --- | --- | --- | --- | --- | --- |
| **Pillar 1: Diagnose** | | | | | | |  |
| 1. Increase access points for HIV testing | | 1. Increase HIV testing availability in CBOs and opt-out testing in EDs and correctional facilities | EHE funds, other private funding sources focused on promoting public health | | TBD, depending on the agency/ organization | |  |
|  |  | 1. Expand HIV self-testing kits access |  |  |  |  |  |
|  |  | 1. Advertise new and existing access points for HIV testing |  |  |  |  |  |
|  |  | 1. Conduct data-driven analysis for expanding HIV testing |  |  |  |  |  |
| ***Pillar 1 Total*** | | | | | ***TBD*** | |  |
| **Pillar 2 : Prevent** | | | | | | |  |
| 1. Increase number of access points for PrEP | | 1. Increase number of CBOs offering PrEP 2. Increasing number of pharmacies dispensing PrEP 3. Increase number of PrEP providers; including primary care providers 4. Identify sources to pay for PrEP costs to achieve free PrEP | Private and public funding sources (TBD), State legislature, OHA prevention program, Pharmacies and Pharmaceutical companies | | $850,000 | | |
| 1. Increase access to HIV Prevention education and increase prevention messaging | | 1. Provide PrEP detailing to increase provider knowledge 2. Develop PrEP educational campaigns 3. Provide age-appropriate comprehensive sex education in schools and other settings targeting young people | Educational organizations, Private and public funding sources, OHA, CDC, HRSA | | $900,000 | | |
| ***Pillar 2 Total (% budget)*** | | | | | ***$1,750,000 (1.8)*** | |  |
| **Pillar 3: Treat** | | | | | | |  |
| 1. Ensure PWH have immediate access to ART | 1. Provide funding to support and modernize ADAP | | HRSA, EHE | | $9,500,000 | |  |
|  | 1. Transition PWH from ADAP to health insurance HICP within 90 days | |  |  | $400,000 | |  |
|  | 1. Employ community health workers or peer counselors to assist with ART linkage in each county | |  |  | $1,280,000 | |  |
| 1. Implement coordinated and comprehensive centralized linkage, retention and re-engagement system | 1. Ensure meaningful involvement of PWH in program design, implementation and quality improvement projects | | HRSA, EHE, Additional philanthropic resources | | $175,000 | |  |
|  | 1. Establish secure, central electronic repository for patient documentation | | HRSA – EHE | | $805,000 | |  |
|  | 1. Provide transportation assistance and food assistance | | Atlanta Food Bank; Atlanta Planning Council; Food Security CBOs;  GA Dept of Transportation | | $24,633,380 | |  |
|  | 1. Strengthen discharge planning from hospitals and correctional institutions | | HRSA – EHE  Additional funders for medications | | $24,633,380 | |  |
|  | 1. Implement culturally reflective electronic application for appointments and services recertifications | | HRSA – EHE | | $375,000 | |  |
|  | 1. Ensure PWH are aware of the services available to them and use geofencing to identify nearest providers | | HRSA – EHE  RWHAP – Part 2 | | $400,000 | |  |
|  | 1. Develop centralized linkage and retention system | | HRSA – EHE | | $19,259,900 | |  |
| 1. Increase the capacity of the workforce | 1. Non-traditional hours by all service providers; incorporate 24-hour drop-in centers | | HRSA – EHE  RWHAP – A | | $8,754,872 | |  |
|  | 1. Non-traditional location of services including co-locating with non-HIV service providers (includes mobile clinics) | | HRSA – EHE  340b rebates | | $1,000,000 | |  |
|  | 1. Equip street teams with telehealth capabilities to serve clients where they are | |  |  | $1,207,575 | |  |
| ***Pillar 3 Total (% budget)*** | | | | | ***$94,424,107 (95.4)*** | |  |
| **Pillar 4: Respond** | | | | | | |  |
| 1. Establish rapid response teams and treat each case as a sentinel event | 1. Create a new system predicated on approach that every new HIV diagnosis represents a sentinel health event for rapid response linkage | | | HRSA, CDC | | $1,160,000 |  |
|  | 1. Embed a surveillance Epidemiologist within Georgia DPH to monitor HIV outbreaks | | | EHE | | $525,446 |  |
| ***Pillar 4 Total (% budget)*** | | | ***$1,685,446 (1.8)*** | | | |  |
| **TOTAL budget** | | | **$96,859,553** | | | |  |

CBOs: community-based organizations; ED: emergency departments; PrEP: pre-exposure prophylaxis; OHA: Office of HIV/AIDS; CDC: Centers for Disease Control and Prevention; HRSA: Health Resources and Services Administration; RWHAP: Ryan White HIV/AIDS Program. Allocations in figure and supporting table adapted from the Ending the HIV Epidemic in Georgia Centers for Disease Control and Prevention EHE Plan; PS 19-1906 (December 2020). Available at: <https://www.cdc.gov/hiv/funding/announcements/ps19-1906/ehe-plans.html> [Accessed June 2, 2022]. Fundings without $ amounts defined were noted as TBD in the EHE plan (see corresponding table for further detail). This table excludes Pillar 5 : Housing and Supportive Services, Pillar 6: Policy, and Pillar 7: Informatics, data analytics and systems cited in the GA 2020 EHE Plan.

**Appendix References**

1. Fulton County Government. Atlanta EMA FY2017 Ryan White Part A Application Narrative 2017 [Available from: <http://www.ryanwhiteatl.org/wp-content/uploads/2016/11/Narrative-FY17-Atlanta-EMA-H89HA00007.pdf>.

2. Association of State and Territorial Health Officials. Profile of State and Territorial Public Health. <https://www.astho.org/Profile/Volume-Four/2016-ASTHO-Profile-Survey-Questionnaire-State/> . Accessed [December 15, 2021].

3. Compendium of Evidence-Based Interventions and Best Practices for HIV Prevention: Centers for Disease Control and Prevention; 2018 <https://www.cdc.gov/hiv/research/interventionresearch/compendium/index.html> . Accessed [December 15, 2021]. [

4. Centers for Disease Control and Prevention: National Prevention Information Network. <https://npin.cdc.gov/> . Accessed [December 15, 2021].

5. Petroll AE, Walsh JL, Owczarzak JL, McAuliffe TL, Bogart LM, Kelly JA. PrEP Awareness, Familiarity, Comfort, and Prescribing Experience among US Primary Care Providers and HIV Specialists. AIDS Behav. 2017;21(5):1256-67.

6. Shea CM, Jacobs SR, Esserman DA, Bruce K, Weiner BJ. Organizational readiness for implementing change: a psychometric assessment of a new measure. Implement Sci. 2014;9:7.
