# Supplementary material for: Ending the HIV Epidemic in Metropolitan Atlanta: a mixed‐methods study to support the local HIV/AIDS response
Source: J Int AIDS Soc. 2024 Jul 22;27(7):e26322. doi: 10.1002/jia2.26322 (PMC11263453; doi:10.1002/jia2.26322)
Supplement: Supplementary file 2 — Additional file 2: “Supplementary Appendix B” Contains supplementary information on study results. [file JIA2-27-e26322-s002.docx]

**SUPPLEMENTARY APPENDIX B**

**Ending the HIV Epidemic in Metropolitan Atlanta: A mixed-methods study to support the local HIV/AIDS response**

Micah Piske^1^, Bohdan Nosyk^1,2§^, Justin C Smith^3,4^, Bianca Yeung^1^, Benjamin Enns^1^, Xiao Zang ^5^, Patrick S Sullivan^6^, Wendy S Armstrong ^7,10^, Melanie A Thompson^8^, Gaea Daniel^9^, Carlos del Rio ^7,10^.

1. Centre for Advancing Health Outcomes, St. Paul’s Hospital, Vancouver, Canada;
2. Faculty of Health Sciences, Simon Fraser University, Burnaby, Canada;
3. Positive Impact Health Centers, Atlanta, USA;
4. Harvard T.H. Chan School of Public Health, Boston, USA
5. Division of Health Policy and Management, School of Public Health, University of Minnesota, Minneapolis, USA;
6. Department of Epidemiology, Emory University, Rollins School of Public Health, Atlanta, USA;
7. Division of Infectious Diseases, Department of Medicine, Emory University School of Medicine, Atlanta, USA;
8. Thacker & Thompson, MD, Atlanta, USA;
9. Nell Hodgson Woodruff School of Nursing, Emory University, Atlanta, USA;
10. Grady Health System, Atlanta, USA.

| **Content** | |
| --- | --- |
| Table B1. Survey response codebook and definitions | 2-4 |

**Table B1.** Response codebook on reported resource needs and barriers in implementing services by EHE Pillar

| **Code** | **Definition** | **Example(s)** |
| --- | --- | --- |
| ***Pillar 1: Diagnosis*** | | |
| HIV testing offer | Provider challenges and attitudes in conducting HIV testing including protocols.  (Note: distinct from testing coverage and funding). | Opt-out testing not routinely offered or prioritized, provider awareness |
| HIV testing coverage | Needs and challenges relating to insurance coverage for HIV testing and costs for clients. | Subsidized resources for rapid tests and self-testing, insurance coverage for routine testing |
| HIV testing funding | Needs relating to organizational funding for HIV testing services and materials. | Funding for testing events in community, provision of free tests and self-testing kits |
| Other testing | Needs and attitudes relating to availability or provision of testing for other concurrent conditions. | STIs, Testing for type 2 diabetes, Hepatitis B and C |
| ***Pillar 2: Prevention*** | | |
| PrEP coverage | Needs and challenges relating to client insurance coverage for PrEP and costs (Note: distinct from availability of providers and funding). | Coverage for PrEP associated lab work, follow-up visits, monitoring fees among uninsured, co-payment and deductible amounts for insured |
| PrEP providers | Needs relating to PrEP accessibility from availability of prescribers. | Number and location of PrEP prescribers, PrEP demand and navigation, PrEP referral mechanisms |
| PrEP funding | Needs relating to organizational funding to offer PrEP and subsidies. | Lack of funding to offer PrEP services in community-based organizations, sustainability of funding mechanisms (CDC and private) |
| Expanded prevention | Needs relating to the scope of HIV prevention services and programs designed for primary prevention. | PrEP resources in testing locations, prevention services funding in county health departments, integrated care beyond testing-only prevention |
| Syringe services | Needs or challenges relating to syringe service programs for injection drug use. | District implementation of syringe service program, funding mechanisms for implementation, syringe service locations |
| ***Pillar 3,4: Treatment, Response*** | | |
| Case management | Needs relating to HIV case management in clinical settings. | Multidisciplinary care linkage and re-engagement, enhanced support, emergency operations center for people newly diagnosed or returning to care, clinical navigation staff for treatment adherence |
| Service demand | Needs and challenges providing services during high demand for treatment services. | Caseloads for patient care navigation, case management support staff, night and weekend clinic hours |
| Drug assistance programs | Needs relating to medication coverage including in Drug Assistance Programs, associated medication coverage and costs. | Expanding range of available medications in ADAP formulary and inclusion process |
| ART access | Needs and challenges relating to accessibility of ART medications for PLWH. | Medication delivery mechanisms, ADAP pharmacy locations and distribution, mobile clinics |
| HIV specialists | Needs relating to accessibility to HIV specialists, availability, support for non-HIV specialists and related resources. | HIV specialists resource and contact list with ratings by location, telehealth HIV specialist consultation, infectious disease collaboration for ART initiation and maintenance |
| Partner services | Needs or challenges relating to services for partners of people newly diagnosed and living with HIV. | Conducting partner service interviews, partner notification, testing, counselling and linkage to care |
| ***Integrated care & social support programs*** | | |
| Sexual health | Needs relating to care integration in sexual health. | HIV-status neutral sexual health clinics, rebranding STI clinics, sexual health promotion |
| Substance use care | Needs relating to scale up or implementation care integration in substance use treatment and support. | Safer injection kits and harm reduction supplies, medications for opioid use disorder |
| Mental health care | Needs relating to scale up or implementation of mental health and counselling services. | Group counselling, expansion of mental health care locations, mobile mental health teams |
| Peer support | Needs relating to scale up or implementation of peer support roles ('Peer' definition provided in survey: a person living with HIV who contributes to positive health outcomes of other community members, but is not usually a health care professional with clinical training) | Peer outreach and care navigation roles in community-based organizations and acute care settings, HIV support groups |
| Housing services | Needs and challenges relating to supportive housing services and related resources for people unstably housed. | HOPWA program challenges, housing support in rural areas, lack of housing for people not eligible for HOPWA |
| Food security | Needs and challenges relating to provision of food and related resources to clients. | Funding for food and nutrition programs, grocery gift cards, pantry services, pantry donations |
| Transportation | Needs or challenges relating to transportation for clients and related access to HIV care. | Travel vouchers, Uber for clients and other transportation options in rural areas, company vans to transport clients |
| Integrated services capacity | Needs relating to capacity constraints in providing integrated care and social support programs. | Funding, human resources and infrastructure for housing support, mental health, transportation in rural areas |
| ***Structural & Policy interventions*** | | |
| Health insurance | Needs and challenges relating to healthcare access and public health insurance policies. | Medicaid coverage gaps, Medicaid expansion, coverage for concurrent conditions, preventative services and testing |
| Involvement of PLWH | Needs relating to meaningful community engagement of PLWH. | Involvement of PLWH in service delivery planning and decision-making |
| Intersectional stigma | Needs relating to addressing racism and stigmatized identities in care settings and other forms of discrimination influencing service delivery and uptake. | Anti-stigma training, LGBTQ cultural awareness, anti-racism and trauma-informed care training for care providers |
| Medical mistrust | Challenges relating to public attitudes and perceptions of medical system influencing participation and connection to care. | HIV misinformation and fear of medical systems, impacts of current and historic racism |
| EHE leadership | Needs and challenges relating to local, regional and state EHE response and activities. | Community-driven EHE response including care providers, stakeholder meetings, directorship vacancies |
| EHE funding | Needs and challenges relating to state funding resources and allocations to support EHE activities. | Increasing state EHE funding for HIV testing and PrEP expansion, funding resources |
| Rural disparities | Needs and challenges in HIV and sexual health care in rural, remote and underserved areas. | HIV/STI care and prevention services expansion outside of downtown core, residential segregation, geographic disparities, health department capacity by county |
| Work conditions | Needs and challenges related to HIV service provider work environments and organizational factors impacting service delivery. | Salaries and benefits for public health staff aligned to cost of living, employee turnover, number of vacancies, recruitment challenges, local and state-level COVID-19 pandemic response |
| HIV education | Needs relating to public education and health promotion on HIV care and prevention. | Provider, agency, community education on HIV prevention and treatment, long-acting injectable PrEP and ART education, youth HIV testing and prevention outreach |

STI: sexually transmitted infection; PrEP: pre-exposure prophylaxis; ADAP: AIDS Drug Assistance Program; ART: antiretroviral therapy; HOPWA: Housing Opportunities for Persons with AIDS; PLWH: people living with HIV; EHE: Ending the HIV Epidemic
